# Hypertrophic Scarring and Keloids: Epidemiology, Molecular Pathogenesis, and Therapeutic Interventions

**DOI:** 10.1002/mco2.70381

**Published:** 2025-10-04

**Authors:** Xiaowan Fang, Yuxiang Wang, Hao Chen, Zhenzhen Yan, Shunxin Jin, Yixin Wu, Futing Shu, Shichu Xiao

**Affiliations:** ^1^ Department of Burn Surgery The First Affiliated Hospital of Naval Medical University Shanghai People's Republic of China

**Keywords:** epidemiology, fibroblast dysregulation, hypertrophic scars, keloids, molecular pathogenesis, scar management

## Abstract

Wound healing is a complex, multicellular process that is essential for restoring tissue integrity after injury. In a subset of individuals, however, this process becomes dysregulated, culminating in hypertrophic scars or keloids—fibroproliferative disorders marked by excessive extracellular matrix deposition and prolonged inflammation. Although these lesions differ clinically, both share overlapping molecular mechanisms involving aberrant activation of the TGF‐β, Intergrin‐FAK, and Wnt/β‐catenin pathways. Recent insights from single‐cell and multiomics technologies have revealed profound heterogeneity within scar‐forming fibroblast populations and highlighted the modulatory roles of immune cells, genetic predispositions, and anatomical tension. However, despite increasing mechanistic understanding, current interventions—including surgery, corticosteroids, and laser therapy—are limited by high recurrence rates and variable efficacy. Emerging strategies now target fibroblast plasticity, inflammatory circuits, and biomechanical feedback via tools such as gene editing, immune modulation, and smart biomaterials. This review integrates advances across epidemiology, molecular signaling, and therapeutic innovation, underscoring the need for personalized, multitargeted approaches. Ultimately, transforming pathological scarring from a persistent clinical burden into a regenerative opportunity will depend on interdisciplinary collaboration and the continued translation of benchside discovery into bedside care.

## Graphical abstract

Hypertrophic scars and keloids highlight distinct epidemiology, multifactorial triggers (genetic, mechanical, medical), and dysregulated signaling hubs (TGF‐β, Integrin‐FAK, Wnt/β‐catenin). Emerging interventions targeting fibroblast plasticity, inflammation, and biomechanics via gene editing, immune modulation, and biomaterials are increasingly favored. Integrating epidemiology, molecular insights, and novel interventions is crucial for developing personalized, multitargeted approaches to transform scarring into regeneration.

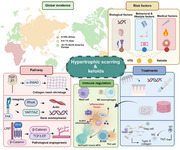

## 1 Introduction

Pathological scarring, including hypertrophic scars (HTS) and keloids, poses a significant clinical and esthetic challenge. These fibroproliferative disorders arise from aberrant wound healing processes and are characterized by excessive collagen deposition, persistent inflammation, and dysregulated fibroblast activity [1]. Unlike normal wound healing, which progresses through controlled inflammatory [2], proliferative, and remodeling phases [3], HTS and keloids exhibit prolonged inflammation and excessive extracellular matrix (ECM) accumulation, culminating in disfiguring and symptomatic scars [4, 5]. Clinically, HTS remains confined to the original wound boundary and may regress over time, whereas keloids invade surrounding tissue, displaying aggressive growth and a high recurrence rate [3].

These pathological scars not only cause functional impairments, such as pain, pruritus, and restricted mobility but also impose a substantial psychosocial burden. Studies have linked scarring to anxiety, depression, and social stigmatization, particularly in highly visible sites [6]. Epidemiological data indicate that more than 70% of burn survivors develop HTS, whereas keloids disproportionately affect individuals of African, Hispanic, or Asian descent, suggesting genetic predisposition and ethnic susceptibility [7]. The annual economic burden of scar management reaches billions of dollars worldwide, attribute to prolonged treatment, surgical revisions, and rehabilitation costs [6].

Current therapeutic strategies for HTS and keloids include corticosteroid injections, surgical excision, laser therapy, and silicone‐based dressings. However, these approaches exhibit variable efficacy, with keloids demonstrating a particularly high recurrence rate [8]. Emerging evidence suggests that mechanical stress, immune dysregulation [2], and genetic predisposition play critical roles in scar progression, yet a comprehensive understanding of the underlying molecular mechanisms remains incompletely characterized [8]. Research has increasingly focused on identifying signaling pathways that govern fibroblast activity and ECM remodeling, such as the transforming growth factor‐beta (TGF‐β), integrin‐focal adhesion kinase (FAK), Wnt/β‐catenin, and Yes‐associated protein/transcriptional coactivator with PDZ‐binding motif (YAP/TAZ) signaling pathways [9]. These mechanistic insights are driving the development of novel therapeutic strategies.

This review provides a comprehensive synthesis of the current understanding of HTS and keloid formation, integrating epidemiological data, molecular pathogenesis, and therapeutic interventions. We begin by outlining the epidemiology of HTS and keloids, including their distribution patterns, risk determinants and anatomical predilection, followed by an in‐depth discussion of the molecular mechanisms underlying pathological scarring, highlighting fibroblast dysregulation, inflammatory mediators, and related signaling pathways. Furthermore, we evaluated both established and emerging therapeutic strategies, including invasive procedures, noninvasive interventions, and novel molecularly targeted treatments. By consolidating recent findings, this review aims to enhance the understanding of HTS and keloid pathophysiology, facilitate the development of improved therapeutic approaches, and identify critical gaps for future research. Through this integrative approach, we seek to provide clinicians, researchers, and healthcare professionals with an up‐to‐date and evidence‐based perspective on HTS and keloid management, ultimately contributing to improved patient outcomes and quality of life.

## 2 Epidemiology

Wound healing is a complex, highly orchestrated process, yet in some individuals, it deviates pathologically, resulting in excessive scar formation. HTS and keloids represent extreme manifestations of dysregulated healing, disproportionately affecting certain populations and anatomical regions. Their occurrence is not random but rather follows distinct epidemiological patterns shaped by genetic, biological, and environmental factors. Understanding these patterns is essential for identifying at‐risk populations and developing targeted prevention and treatment strategies.

### 2.1 Distribution Patterns

#### 2.1.1 Geographic Trends

**FIGURE 1:**
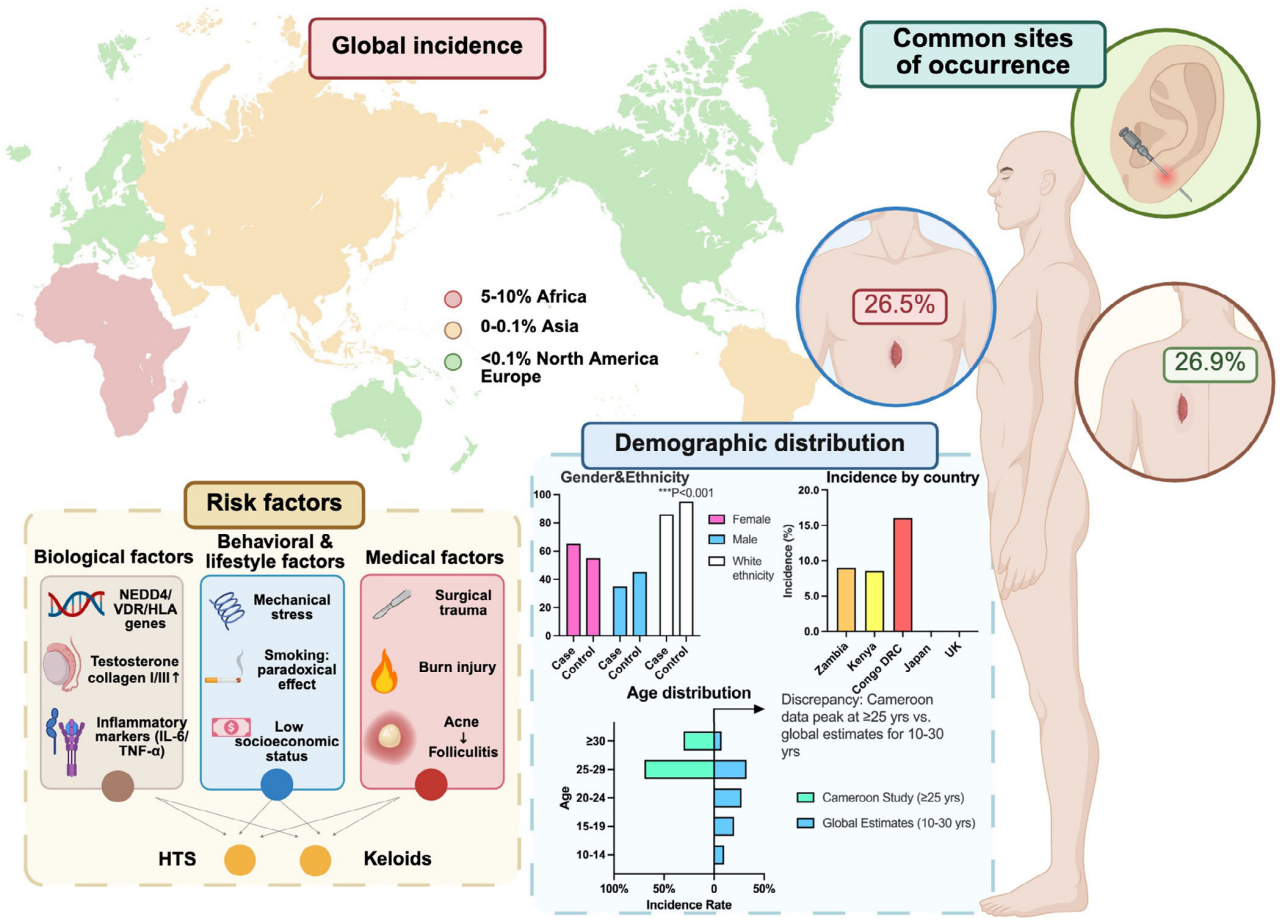
Illustrative summary of epidemiological characteristics. This figure provides a visual overview of the key epidemiological findings discussed in the text. The top‐left panel shows a heatmap of global incidence rates, highlighting a high prevalence in African populations (5%–10%, e.g., Cameroon: 3.5%), with lower rates in Asian (e.g., Japan: 0.1%) and Western countries (e.g., UK: 0.09%). The bottom‐left panel summarizes major risk factors, grouped into biological (e.g., HLA‐DRB1*15), behavioral (e.g., mechanical stress in high‐tension regions), and medical (e.g., postsurgical trauma) categories. The middle‐bottom panel presents key demographic patterns, including age (peak: 10–30 years), sex, and ethnicity trends. The right panel includes a 3D anatomical diagram identifying common sites of keloid formation, such as the anterior chest (26.5%) and scapular area (26.9%). This integrative depiction supports the complex interplay of genetic, environmental, and biomechanical factors in scar pathogenesis. Created in https://BioRender.com.

The prevalence of HTS and keloids varies substantially across different geographic regions, as illustrated in Figure 1. Studies indicate a higher incidence of keloids in equatorial regions, particularly among African (5%–10%, with Cameroon reporting 3.5%), Asian (0%–0.1%, Japan: 0.1%) [10], and Latin American populations, whereas lower rates are observed in European and North American populations (< 0.1%, UK: 0.09%) [11]. This geographic disparity is largely attributed to genetic predispositions, as well as to environmental and lifestyle factors, including sun exposure and differences in healthcare access. Intracountry variations may exist, with potential differences between urban and rural populations due to factors such as disparities in access to healthcare, socioeconomic status, and occupation‐related exposures. However, the extent of these differences remains uncertain and may vary by context [12, 13].

#### 2.1.2 Demographic Characteristics

The development of HTS and keloids is influenced by multiple demographic factors, including age, sex, ethnicity, and occupation.

Keloids most frequently occur in individuals aged 10–30 years, with a peak incidence during puberty and early adulthood, as shown in Figure 1. This age‐related pattern suggests a potential link between hormonal fluctuations and abnormal scar formation.

Sex differences in scar formation have been inconsistently reported. While some studies indicate a female predominance, possibly due to greater esthetic concern and a greater likelihood of seeking medical attention, a recent study of Chinese college students identified male sex as a significant risk factor for HTS (adjusted *p* < 0.001) [14]. Furthermore, estrogen has been proposed to modulate fibroblast activity and collagen synthesis, potentially contributing to sex‐based differences in scar formation. Recent research has also demonstrated that androgenic steroids, such as testosterone, drive pathological scarring. In a preclinical porcine model, testosterone treatment significantly increased the scar area and thickness and was associated with increased cellular metabolism and immune response gene sets. Scars from testosterone‐treated animals presented greater collagen deposition, particularly Types I and III, which may explain their enhanced mechanical properties [15].

Ethnicity plays a pivotal role in keloid incidence, with markedly higher incidence rates observed in darker‐skinned individuals, particularly those of African, Asian, and Hispanic descent. The underlying mechanisms are believed to involve genetically determined differences in fibroblast behavior, ECM composition, and inflammatory responses [12], as highlighted in Figure 1.

Occupation is another critical factor, as individuals engaged in professions involving frequent skin trauma, mechanical stress, or exposure to hazardous environments (e.g., manual laborers, military personnel) may have an elevated risk of developing HTS.

### 2.2 Risk Determinants

#### 2.2.1 Biological Factors

Several biological factors contribute to the development of HTS and keloids. Genetic predisposition is a well‐established risk factor, with familial aggregation frequently observed. Specific major histocompatibility complex (MHC) alleles, such as HLA‐DRB1*15 (OR = 1.68) [27], and polymorphisms in genes such as NEDD4 and the vitamin D receptor (VDR) [16] have been associated with increased susceptibility across multiple populations [17, 18, 19]. Additionally, individuals with a personal or familial history of keloids or HTS exhibit a heightened risk of developing subsequent lesions, suggesting a hereditary component [20].

Immune dysregulation also plays a pivotal role in pathological scarring. Conditions such as atopic dermatitis and other chronic inflammatory skin disorders have been linked to an elevated risk of keloid development, likely due to prolonged inflammation and dysregulated wound‐healing responses [21]. Dysregulated cytokine signaling, persistent immune cell infiltration, and aberrant macrophage polarization may maintain a profibrotic microenvironment, sustaining fibroblast activation and collagen overproduction. Although several susceptibility loci have been identified, no current genetic test can reliably predict keloid risk, highlighting the need for validation studies in larger, multiethnic cohorts.

#### 2.2.2 Behavioral and Lifestyle Influences

Behavioral and lifestyle factors significantly impact the risk of developing HTS and keloids. Socioeconomic status has been identified as a contributing factor, as individuals from lower‐income backgrounds may have limited access to early wound care, preventive measures, and advanced therapeutic options, increasing their susceptibility to excessive scar formation [22].

Mechanical stress is a critical determinant of scar development. Wounds located in high‐tension areas, such as the shoulders, anterior chest, and joints, are more likely to develop into HTS or keloids because of repetitive movements and mechanical forces [23], as depicted in Figure 1. This phenomenon highlights the role of mechanotransduction, wherein fibroblasts respond to mechanical stimuli by altering collagen deposition and ECM remodeling.

Interestingly, smoking has been proposed to have a paradoxical effect on scar formation. Some studies suggest that nicotine‐induced vasoconstriction may reduce fibroblast proliferation and collagen synthesis, potentially leading to a lower incidence of HTS [24, 25]. However, this association remains inconclusive and requires further investigation.

#### 2.2.3 Medical Triggers

Surgical and traumatic injuries are common initiating events in HTS and keloid development, particularly when they involve high‐tension closures or delayed healing. Examples include sternotomies, orthopedic procedures, and severe burns, all of which are associated with extensive dermal damage, prolonged inflammation, and persistent fibroblast activation. Acne‐related dermal inflammation is another frequently reported nonsurgical cause, especially in predisposed individuals. These medical insults induce sustained inflammatory microenvironments, which drive abnormal collagen synthesis and ECM deposition [26].

### 2.3 Anatomical Predilection

reHTS and keloids predominantly develop in anatomical regions subjected to mechanical stress and high skin tension, as illustrated in Figure 1. The presternal region (anterior chest) is the most frequently affected site (26.5% prevalence [27]), particularly among young males, and often results from surgical incisions or traumatic injuries. The torso and back are also commonly affected, with scar formation influenced by repetitive stretching, friction, and mechanical forces. The scapular region is another high‐risk area (26.9% prevalence [10]), where shoulder mobility exacerbates mechanical tension, increasing the likelihood of HTS [28, 29, 30]. Although extremities are less commonly affected, they remain an important site for HTS and keloids, particularly following burns, trauma, or surgical procedures [31].

Although traditionally considered a low‐tension site, the earlobe is particularly prone to keloid formation, primarily due to piercing‐induced dermal trauma and its unique fibroblast behavior [32]. Unlike regions where mechanical stretching predominates, earlobe keloids exhibit dense deposition of hyalinized keloidal collagen (HKC), which begins accumulating around perivascular areas as early as three months postinjury and progresses into interconnected “bitten donut”‐like structures in chronic lesions [33]. This time‐dependent matrix remodeling is not correlated with lesion size, suggesting intrinsic fibroblast dysregulation rather than tension‐dependent expansion. Histologically, fibroblasts isolated from earlobe keloids show increased metabolic activity and increased collagen and proteoglycan synthesis. Recent scRNA‐seq analyses revealed that Schwann cells (SCs) in earlobe tissue engage in pathological crosstalk with mesenchymal fibroblasts through the SEMA3C–NRP/PLXND1 signaling axis, promoting fibroblast proliferation and ECM remodeling. Fibroblasts, in turn, enhance SC migration via MK and PTN ligand expression, forming a feedback loop that sustains keloid growth [34]. Additionally, mast cell degranulation, frequently observed in giant earlobe keloids, releases tryptase and histamine, which activate fibroblasts through PAR2 receptors, further exacerbating collagen deposition [35]. These findings imply that even at sites of low mechanical tension, molecular and cellular microenvironmental cues can independently drive keloid pathogenesis.

Therefore, identifying both high‐tension regions and sites with inherent cellular susceptibilities, such as the earlobe, is crucial for early risk stratification and tailored therapeutic approaches.

## 3 Molecular Pathogenesis

**FIGURE 2:**
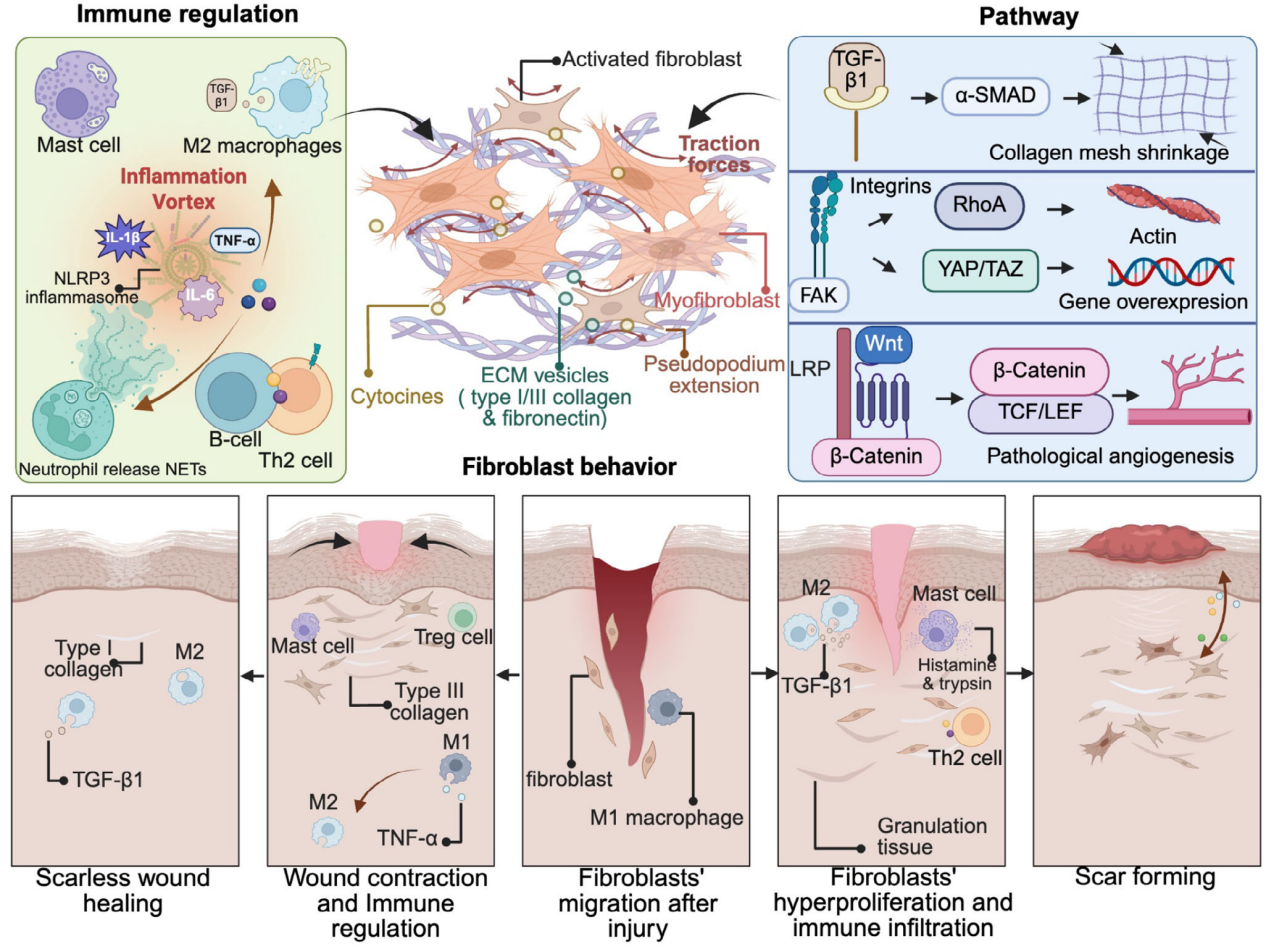
Schematic diagram illustrating the pathogenesis of hypertrophic scars and keloids. This figure delineates the molecular and cellular mechanisms driving pathological scarring. In normal wound healing, balanced immune regulation promotes transient fibroblast activation, controlled collagen remodeling (transition from Type III to Type I), and timely apoptosis of myofibroblasts, ensuring scar‐free repair. M1 macrophages transition to reparative M2 phenotypes, whereas regulatory T cells (Tregs) suppress excessive inflammation. In contrast, HTS and keloids arise from dysregulated immune‐fibroblast crosstalk. Dominant M2 macrophages, mast cells (releasing histamine and tryptase), and Th2 cells (secreting IL‐4/IL‐13) sustain a profibrotic milieu via TGF‐β1 overproduction and NLRP3 inflammasome activation. Neutrophil extracellular traps (NETs) and a cytokine storm (elevated IL‐1β, IL‐6, TNF‐α, CCL2, and CXCL8) further amplify inflammation. Fibroblasts in stiff, disordered collagen matrices exhibit pathological mechanoresponses: integrin αVβ1 clustering activates FAK–YAP/TAZ signaling, driving proliferation, migration, and persistent myofibroblast differentiation. Concurrent Wnt/β‐catenin pathway dysregulation disrupts ECM homeostasis, whereas aberrant TGF‐β signaling (via Smad2/3 and noncanonical MAPK/PI3K cascades) synergizes with mechanical stress to exacerbate collagen overproduction. Created in https://BioRender.com.

Understanding the molecular pathogenesis of HTS and keloids requires a comprehensive view of the cellular and signaling disruptions that distinguish pathological scarring from normal wound healing. While physiological repair involves a tightly regulated sequence of inflammatory resolution, fibroblast activation, matrix remodeling, and tissue regeneration, HTS and keloids reflect a breakdown in these regulatory checkpoints. Central to this dysregulation are sustained immune–fibroblast interactions, aberrant mechanotransduction signaling, and chronic inflammatory cues that perpetuate fibrosis and tissue dysfunction. Recent research has revealed that pathological scars are not simply an exaggeration of normal healing but represent a distinct biological state underpinned by complex, self‐reinforcing molecular networks. These networks encompass key signaling axes, such as the TGF‐β/Smad, Wnt/β‐catenin, and FAK‐YAP/TAZ axes, which together drive fibroblast proliferation, persistent myofibroblast differentiation, and excessive ECM deposition. Moreover, the role of immune cells—particularly M2 macrophages, mast cells, and Th2 lymphocytes—in creating a profibrotic milieu has emerged as a critical driver of chronic scarring. These intertwined processes are illustrated in the schematic overview of fibrotic progression and immune–stromal dysregulation presented in Figure 2, which highlights the key pathogenic events underlying HTS and keloid formation.

### 3.1 Diagnostic and Pathobiological Foundations

Although HTS and keloids both present clinically as firm, elevated, erythematous, or hyperpigmented lesions accompanied by pruritus and pain, they exhibit fundamental differences in their growth behavior. HTS are typically confined to the original wound margins and tend to regress over time, whereas keloids often extend beyond the wound boundaries, continue to expand, and display a high recurrence rate, suggesting a more complex underlying pathobiology [6, 9, 36]. These clinical distinctions are mirrored histopathologically in terms of ECM deposition patterns, cellular composition, and inflammatory activity. In HTS, collagen fibers are commonly arranged in parallel bundles, accompanied by increased expression of α‐smooth muscle actin (α‐SMA), which is indicative of myofibroblast‐mediated contraction [37]. In contrast, keloids are characterized by disorganized and densely packed “keloidal collagen,” with relatively low α‐SMA expression, suggesting a limited role for myofibroblasts in their progression [38]. Moreover, keloids feature a persistently inflamed microenvironment, with sustained infiltration of macrophages, mast cells, and T lymphocytes, along with elevated pro‐inflammatory cytokines such as IL‐1β, IL‐6, and TNF‐α. These findings support the hypothesis that keloids represent a chronic inflammatory skin disorder [39, 40, 41].

Thus, these clinical and histological differences provide diagnostic clues that reflect distinct pathobiological foundations, primarily involving dysregulated fibroblasts, uncontrolled fibrosis, and different molecular signatures.

#### 3.1.1 Fibroblast Activation and Dysregulation in Wound Healing

Fibroblasts participate in all stages of wound healing, and their dysregulation can drive fibrotic outcomes. During the hemostasis phase, platelets accumulate at the injury site and release cytokines, including TGF‐β, which initiate fibroblast recruitment and activation. This primes the wound bed for subsequent cellular responses. In the inflammatory phase, macrophages play a key role in clearing necrotic tissue and secreting profibrotic factors that modulate fibroblast behavior. Subsets of macrophages (e.g., CD206⁺/CD301b⁺) promote the differentiation of fibroblasts into myofibroblasts, whereas mast cell–derived histamine and tryptase increase fibroblast proliferation and ECM production.

During the proliferative phase, fibroblasts migrate into the wound bed, deposit ECM components, and differentiate into α‐SMA–positive myofibroblasts to increase contractile activity and collagen deposition. However, in keloids, the myofibroblast population is reduced, suggesting that their expansion relies less on contraction and more on aberrant ECM remodeling and immune‐driven signaling [37, 38, 39]. Moreover, fibroblasts engage in crosstalk with keratinocytes, where keratinocyte‐derived IL‐1 and TNF‐α stimulate fibroblasts to secrete keratinocyte growth factor (KGF), supporting reepithelialization. Disruption of this reciprocal interaction impairs epidermal regeneration and promotes fibrotic remodeling, especially under conditions of mechanical loading.

Normally, during the remodeling phase, fibroblasts undergo apoptosis or return to a quiescent state to restore tissue homeostasis. They also regulate ECM turnover by producing enzymes such as MMPs and matricellular proteins that coordinate collagen remodeling. For example, galectin‐1 modulates cell–matrix interactions and fibroblast migration, whereas macrophage‐derived proteases facilitate the transition from early Type III collagen to mature Type I collagen. However, in pathological scars, this resolution fails, leading to persistent fibroblast activation and excessive collagen production. This phenomenon is especially pronounced in keloids and may be driven by epigenetic regulation, cytokine dysregulation, and feedback from mechanical stress [40, 42]. Recent studies have indicated that CD74⁺ fibroblasts in keloids are particularly mechanosensitive, responding to tensile stress with increased proliferation and sustained fibrotic signaling [43, 44, 45].

#### 3.1.2 ECM Remodeling and Tissue Dysfunction in HTS and Keloids

The pathological transformation of fibroblasts in HTS and keloids leads to persistent ECM deposition and metabolic dysregulation. Unlike HTS, where fibroblast overactivity is self‐limiting, keloid fibroblasts evade apoptosis and maintain continuous fibroblast activation, sustaining the accumulation of ECM components, particularly Type I collagen, and promoting abnormal tissue expansion beyond the original wound margins. In keloid fibroblasts, genetic and epigenetic alterations underlie this sustained fibrotic activity. Evidence from the literature indicates that single‐nucleotide polymorphisms (SNPs) in the promoter region of COL1A1 (e.g., rs1800012) increase transcription factor binding and upregulate Type I collagen expression [46]. Notably, this SNP has been identified by sequencing analyses of keloids and is correlated with the excessive collagen deposition phenotype characteristic of fibroblasts in keloid lesions. Additionally, SNPs in TGFBR1 (e.g., rs334348) increase Smad2/3 phosphorylation and promote fibroblast‐to‐myofibroblast differentiation [47], a transition widely observed in the hypercontracting and fibrotic phenotypes of fibroblasts isolated from HTS and keloids. Copy number amplifications of VEGFR2 and FGFR1/2 further activate the PI3K/AKT and MAPK pathways, enhancing fibroblast proliferation, collagen synthesis, and angiogenesis [48]. These alterations have been reported in transcriptomic and genomic studies of HTS and keloid tissues, supporting a pathogenic model in which genetic and epigenetic changes in fibroblasts drive sustained fibrogenesis and aberrant tissue remodeling. Epigenetic dysregulation also reinforces the profibrotic phenotype. Hypomethylation of the TIMP2 promoter decreases MMP activity and impairs ECM degradation [48], whereas histone modifications such as H3K27ac enrichment at the CTGF and PAI‐1 enhancer regions maintain open chromatin and promote transcription [49]. In addition, noncoding RNAs such as miR‐29a‐3p, which normally represses COL1A1 and TGFBR1, are downregulated in keloids, whereas competing lncRNAs (e.g., H19) and circRNAs (e.g., hsa_circ_0020792) sequester miRNAs to further activate TGF‐β/Smad signaling and collagen expression [46, 50, 51, 52, 53]. These multilevel regulatory changes sustain abnormal fibroblast behavior and ECM remodeling.

Emerging evidence suggests that epithelial‐to‐mesenchymal transition (EMT) in keratinocytes and endothelial‐to‐mesenchymal transition (EndMT) in vascular endothelial cells further amplify fibroblast‐driven fibrosis. EMT refers to a biological process in which epithelial cells, such as keratinocytes, lose their apical–basal polarity and epithelial junctions, acquiring mesenchymal characteristics, including enhanced motility and ECM‐producing capacity. Similarly, EndMT is a parallel phenotypic shift occurring in endothelial cells, whereby they adopt fibroblast‐like features and contribute to pathological tissue remodeling. Both EMT and EndMT generate a substantial pool of activated fibroblasts—particularly myofibroblasts—which are key effectors of ECM overproduction in fibrotic conditions. Numerous studies have documented the involvement of these transitions in organ fibrosis, including kidney, cardiac, and ocular fibrosis.

In the context of cutaneous fibrosis, such as HTS and keloids, EMT and EndMT have also been implicated. For example, keratinocytes in keloids exhibit EMT‐like phenotypes characterized by elevated collagen Iα1, fibronectin, and α‐SMA expression, whereas PDE4B overexpression promotes TGF‐β1‐induced mesenchymal transformation—effects that are reversed by PDE4 inhibition [53]. Similarly, EndMT has been identified as a source of myofibroblasts in scar tissue, with up to 84% of endothelial cells coexpressing α‐SMA in HTS [54]. Artesunate, a potential antifibrotic agent, has been shown to attenuate EndMT and reduce HTS formation by inhibiting both the PI3K/AKT/mTOR pathway and the TGF‐β/Smad pathway [55]. TGF‐β is a principal inducer of EMT and EndMT through canonical Smad signaling, reinforcing fibrotic progression. The therapeutic potential of targeting these pathways, particularly through ECM modulation, will be further discussed in Section 4.2.

#### 3.1.3 Fibroblast Heterogeneity and Single‐Cell Insights

Recent advances in single‐cell multiomics technologies have revealed the remarkable heterogeneity of fibroblasts, shedding light on their diverse roles in wound healing and pathological scarring [56]. These studies have identified distinct fibroblast subpopulations and cellular states that contribute to either regenerative or fibrotic outcomes, providing new insights into the mechanisms underlying HTS and keloids. For example, single‐cell RNA sequencing (scRNA‐seq) revealed that dermal fibroblasts exhibit lineage‐specific behaviors during wound repair, with extrafollicular progenitors marked by the quiescence‐associated factor Hic1 playing a pivotal role in mediating regeneration or scar formation depending on their spatial localization within the wound [57]. Similarly, cross‐tissue fibroblast atlases have identified universal fibroblast transcriptional subtypes that are conserved across species and tissues, highlighting their potential as reservoirs for specialized fibroblasts in both homeostasis and disease [58]. These findings underscore the importance of fibroblast plasticity in determining wound healing outcomes and suggest that dysregulation of specific fibroblast subpopulations may drive pathological scarring [59].

Moreover, single‐cell analyses have revealed novel fibroblast progenitors and their regulatory programs, which are critical for orchestrating tissue repair. For example, CD201^+^ fascia progenitors have been shown to generate pro‐inflammatory fibroblasts and myofibroblasts in a spatiotemporally controlled manner, with retinoic acid and hypoxia signaling acting as key checkpoints in their differentiation [60]. Disruption of these pathways can impair wound healing, emphasizing the functional importance of fibroblast heterogeneity in maintaining tissue integrity. Additionally, studies have identified a subset of fibroblasts with myeloid origins that contribute to adipocyte regeneration during wound healing, further expanding our understanding of fibroblast diversity and its implications for tissue repair [61]. These discoveries highlight the complex interplay between fibroblast subpopulations and their microenvironment, which can either promote regeneration or drive fibrosis.

The identification of Lef1‐expressing fibroblasts as transient regenerative cell types in neonatal skin has also provided critical insights into the mechanisms of scarless wound healing [62]. These fibroblasts, characterized by their expression of canonical Wnt signaling components, prime the adult skin macroenvironment to enhance repair and regeneration, suggesting potential therapeutic targets for promoting scarless healing in adults. Collectively, these single‐cell studies have revealed the intricate regulatory networks governing fibroblast behavior and their contributions to pathological scarring. By elucidating the molecular and cellular mechanisms underlying fibroblast heterogeneity, these findings pave the way for developing targeted therapies that modulate fibroblast activity and improve wound healing outcomes in HTS and keloids.

### 3.2 Signaling Pathway Advances

#### 3.2.1 Mechanotransduction Signaling

HTS and keloids are both characterized by excessive fibroblast activation and ECM deposition [63], processes that are tightly regulated by mechanical cues within the wound microenvironment. Recent studies have identified several mechanosensitive and fibrogenic signaling pathways that orchestrate these pathological responses. Among them, the TGF‐β, Integrin‐FAK, and YAP/TAZ signaling pathways are considered core mediators because of their high sensitivity to biomechanical stress and their capacity to transduce mechanical stimuli into fibrotic cellular responses. In addition to these canonical pathways, other pathways, such as the PI3K/Akt, Rho GTPase, and ion channel–related signaling cascades, also contribute to fibroblast migration, cytoskeletal remodeling, and ECM dynamics. These pathways often exhibit extensive crosstalk, amplifying fibrotic signaling and reinforcing the chronic activation of myofibroblasts [64]. Together, they form a complex regulatory network that governs the mechanical and biochemical interactions underlying pathological scar formation.

##### 3.2.1.1 TGF‐β Signaling

The TGF‐β signaling pathway is a critical regulator of fibrotic responses and plays a central role in HTS and keloid pathogenesis. Upon ligand binding, TGF‐β engages Types I and II serine/threonine kinase receptors, which activate downstream Smad2/3 proteins. These phosphorylated Smads complex with Smad4 and translocate to the nucleus to drive the transcription of profibrotic genes, such as COL1A1, COL3A1, and ACTA2 (α‐SMA) [63]. In addition to this canonical Smad‐dependent route, TGF‐β also activates several noncanonical pathways—namely, the MAPK (ERK, JNK, p38), PI3K/Akt, and Rho GTPases—that further increase fibroblast proliferation, migration, and cytoskeletal remodeling [64, 65]. Together, these cascades contribute to myofibroblast differentiation and excessive ECM accumulation, which are hallmarks of pathological scarring.

As a classical profibrotic pathway, TGF‐β signaling is tightly modulated by mechanical stress. Both matrix stiffness and tensile forces, which are elevated in fibrotic lesions, promote sustained TGF‐β1 activation and reinforce the fibroblast–myofibroblast transition [66]. In vitro, fibroblasts cultured on rigid substrates display increased TGF‐β signaling activity, whereas in vivo studies using stiff biomaterials have confirmed localized fibroblast proliferation and ECM overproduction. Dynamic forces, including shear stress and cyclic stretch, also upregulate the expression of TGF‐β and its downstream mediators, leading to dermal thickening and persistent remodeling [67, 68] Importantly, pharmacologic blockade of TGF‐β1 under these biomechanical conditions has been shown to suppress collagen synthesis and the persistence of myofibroblasts without compromising local immune surveillance [69].

In addition to mechanotransduction, nonmechanical stimuli can also activate the TGF‐β pathway and contribute to fibrogenesis. Epigenetic regulators, such as histone deacetylase 5 (HDAC5), inhibit Smad7—a key negative regulator of TGF‐β signaling—and thereby prolong fibrotic activation. Targeted inhibition of HDAC5 restores Smad7 expression and significantly attenuates scar formation [70]. Certain phytochemicals, such as isorhamnetin (ISO), reduce TGF‐β‐mediated Smad phosphorylation and fibroblast contractility, suggesting potential therapeutic effects [75]. Other upstream molecules, including integrin β1 (ITGB1), function as mechanosensitive activators of latent TGF‐β1; pharmacologic inhibition via agents such as crizotinib has been shown to reduce dermal thickening in scar models [71]. Emerging modulators such as tanshinone IIA (TSA), the TWEAK/Fn14 axis, and SDPR have also been reported to interfere with TGF‐β activation and collagen production, further highlighting the complexity of its regulation [72, 73, 74]. These findings suggest new avenues for therapeutic intervention; specific strategies are discussed in Section 4.3.

In summary, TGF‐β signaling acts as a central integrator of fibrotic cues, with its activation driven by both mechanical and biochemical stimuli. These multilayered regulatory mechanisms underscore the importance of TGF‐β as a therapeutic target and highlight the need for combinatory strategies for effective scar modulation.

##### 3.2.1.2 Integrin–FAK Axis

The integrin–FAK signaling pathway is a fundamental mediator of mechanotransduction, regulating cellular responses to extracellular mechanical stimuli and playing a pivotal role in fibrotic tissue remodeling, such as in HTS and keloids [75]. Integrins, heterodimeric transmembrane receptors composed of α and β subunits, act as mechanosensors by physically linking the actin cytoskeleton to the ECM [76]. This mechanical linkage facilitates processes such as cellular adhesion, migration, and contractility under tensile stress.

Upon mechanical stimulation, conformational changes in integrin β subunits induce receptor clustering and recruit FAK to focal adhesions, where it undergoes autophosphorylation at Tyr397. This initiates downstream activation of the PI3K/Akt, ERK, and mTOR pathways, which promote fibroblast proliferation, myofibroblast differentiation, and collagen deposition, thereby sustaining a profibrotic microenvironment [77, 78, 79]. In fibrotic scar tissues, the upregulation of the integrin β1 and αV subunits is correlated with increased cellular adhesion and ECM production. Notably, activated FAK also enhances TGF‐β1 secretion, establishing a positive feedback loop with the TGF‐β/Smad axis that reinforces persistent fibroblast activation and fibrogenesis. Targeted inhibition of the integrin–FAK axis in scar‐derived myofibroblasts has been shown to reduce the expression of fibrosis‐related genes such as SMYD3 and ITGBL1, promoting myofibroblast dedifferentiation and attenuating collagen accumulation [80]. These findings underscore the role of the integrin–FAK axis not only as a mechanosensor but also as a key regulator of fibroblast phenotypic plasticity. Preclinical models demonstrate that pharmacological blockade of FAK or upstream integrins can mitigate fibrosis—therapeutic implications are discussed in Section 4.3 [81]. ECM regulators further modulate this axis. For example, lumican—a small leucine‐rich proteoglycan—binds integrins and focal adhesion components to negatively regulate fibrotic signaling. Lumican is essential for collagen fibrillogenesis and normal matrix architecture. However, its expression is significantly downregulated in HTS tissue, partly due to TGF‐β1–mediated suppression [82]. Restoration of lumican expression in vitro inhibits fibroblast proliferation and ECM overproduction, indicating its potential as an upstream regulator of integrin–FAK signaling [83].

In addition, transcriptional regulators such as Snail‐1, which governs EMT and EndMT, have been implicated in modulating this signaling axis. In human dermal microvascular endothelial cells, Snail‐1 overexpression enhances FAK and integrin phosphorylation, promotes focal adhesion reorganization, and increases the expression of ECM components, including collagen I/III/VI and fibronectin [84]. While not a classical mechanotransduction factor, Snail‐1 contributes to the expansion of profibrotic cell populations and may act synergistically with integrin–FAK signaling.

Collectively, the integrin–FAK axis emerges as a central hub linking mechanical stimuli to fibrotic outcomes in HTS and keloids. Its crosstalk with TGF‐β signaling, modulation by ECM components such as lumican, and integration with transcriptional programs such as Snail‐1–driven EMT highlight multiple nodes of therapeutic intervention. Targeting this pathway may offer a promising strategy to disrupt the fibrotic cascade and improve scar remodeling.

##### 3.2.1.3 YAP/TAZ

YAP and transcriptional coactivators with PDZ‐binding motifs (TAZs) are essential mechanosensitive transcriptional regulators that translate extracellular physical stimuli into gene expression programs. As downstream effectors of the Hippo signaling pathway, YAP/TAZ dynamically respond to ECM stiffness, cytoskeletal tension, and cellular geometry, thereby regulating fibroblast proliferation, differentiation, and matrix remodeling, which are pivotal in normal tissue repair. Studies have shown that fibroblast‐specific deletion of YAP/TAZ in murine models results in impaired wound closure and diminished fibrosis, highlighting their indispensable role in cutaneous repair. However, persistent activation of YAP/TAZ in fibrotic skin is strongly associated with pathological remodeling. Studies have shown that YAP/TAZ is significantly upregulated in response to mechanical stress in HTS fibroblasts (HSFs), exacerbating scar formation by driving cell survival, collagen deposition, and matrix contraction.

Evidence has shown that mechanical cues from the ECM are primarily sensed through integrins, which activate the RhoA/ROCK pathway and facilitate actin polymerization via talin and FAK. Myosin II‐generated contractile forces then transmit mechanical stress to the nucleus, leading to the nuclear translocation of YAP/TAZ and the subsequent activation of fibrogenic genes such as CTGF and Col1a1 [85]. Under conditions of high ECM stiffness, actomyosin tension inhibits the Hippo kinase cascade, preventing LATS1/2‐mediated phosphorylation of YAP/TAZ and allowing their accumulation in the nucleus. This promotes fibroblast proliferation, contractility, and excessive ECM production [86]. Conversely, in low‐stiffness environments, the small GTPase RAP2 acts as a mechanosensor that activates LATS1/2 kinases, restoring Hippo pathway activity and resulting in YAP/TAZ inactivation through cytoplasmic sequestration [87, 88]. These findings suggest that fibroblasts interpret mechanical signals through a finely tuned Hippo–YAP/TAZ axis, which plays a critical role in determining fibrotic outcomes.

Moreover, YAP/TAZ signaling closely interacts with other key profibrotic pathways, including the TGF‐β and Wnt/β‐catenin pathways, forming a coordinated mechanical‒transcriptional regulatory network that reinforces fibroblast activation in stiffened, fibrotic tissue microenvironments.

Recent studies on the YAP/TAZ pathway have revealed its critical role in promoting fibroblast activation, ECM accumulation, and myofibroblast differentiation in HTS and keloids. These findings underscore YAP/TAZ as central effectors of mechanotransduction and key drivers of pathological fibrosis. Therapeutically, they offer a compelling target for modulating fibrosis while preserving normal repair. Specific treatment strategies and translational applications are discussed in greater detail in Section 4.3.

##### 3.2.1.4 Additional Mechanotransduction Pathways

In addition to the TGF‐β, integrin–FAK, and YAP/TAZ signaling pathways, several other mechanotransduction cascades—including the PI3K/Akt, Rho GTPase, and ion channel pathways—respond to mechanical stimuli and contribute to fibroblast activation, ECM accumulation, and sustained fibrosis in HTS and keloids.

The PI3K/Akt pathway is a key regulator of cell proliferation, metabolism, migration, and ECM synthesis, and its activation enhances cellular sensitivity to mechanical stimuli. Under mechanical stress conditions, phosphoinositide 3‐kinase (PI3K) and Akt are significantly upregulated, promoting fibrosis‐associated processes [89, 90]. Studies on HaCaT keratinocytes and human HSFs have shown that exposure to micronegative pressure leads to elevated AKT1 and PI3K levels, which peak within 4–12 h and remain sustained [91]. This pathway also interacts with the mTOR signaling cascade to drive inflammation, angiogenesis, and excessive ECM production [92]. Notably, pharmacological inhibition of PI3K/Akt has been explored as a potential strategy to mitigate stretch‐induced hypertrophic scarring.

The Rho GTPase signaling pathway is another key mechanotransduction pathway involved in fibrosis. Rho GTPases function as molecular switches that regulate cytoskeletal remodeling, fibroblast proliferation, and ECM deposition. Among them, RhoA and its downstream effector, Rho‐associated protein kinase (ROCK), are highly expressed in fibrotic tissues, where they promote scar contracture through actomyosin‐mediated tension. Increased RhoA/ROCK activity has been implicated in mechanically induced fibrosis, whereas experimental inhibition of this pathway has been shown to reduce scar thickness and improve wound healing outcomes [93, 94]. These findings suggest that RhoA/ROCK inhibitors may offer therapeutic benefits for fibrosis‐related tissue contracture.

Mechanosensitive ion channels, particularly transient receptor potential (TRP) channels, mediate cellular responses to mechanical and chemical stimuli by modulating calcium influx. Different TRP subfamilies influence fibroblast proliferation, migration, and differentiation and collagen deposition [95]. Research indicates that blocking calcium ion influx can prevent mechanically induced cytoskeletal reorganization and fibrotic progression [91]. A summary of TGF‐β and other mechanosensitive pathways is shown in Table 1. Moreover, the inhibition of mechanosensitive ion channels suppresses PI3K/Akt activation, leading to cytoskeletal depolymerization and reduced fibroblast contractility under mechanical stress.

The involvement of these additional pathways in mechanotransduction underscores their potential as therapeutic targets in fibrosis management. The interplay among the PI3K/Akt, Rho GTPase, and ion channel pathways suggests that targeting mechanosensitive signaling could lead to novel antifibrotic interventions. Future research should focus on delineating the crosstalk between these pathways and identifying precise therapeutic strategies for hypertrophic scarring and keloid prevention.

**TABLE 1:** Mechanosensitive signaling pathways implicated in HTS and keloid formation: recent advances in molecular mechanisms, research models, and therapeutic targets.

Pathway	Key findings	Research models	Potential therapeutic targets	References
**TGF‐β**	‐ Central regulator of fibrosis via Smad2/3 (canonical) and MAPK, PI3K/Akt, Rho GTPases (noncanonical) pathways. ‐ Mechanical stress (e.g., matrix stiffness) enhances TGF‐β1 activation and myofibroblast transition. ‐ Epigenetic regulators (e.g., HDAC5) suppress Smad7; integrins (e.g., ITGB1) activate latent TGF‐β1.	In vitro fibroblasts (rigid substrate), stiff biomaterial mouse models	‐ Anti‐TGF‐β1 antibodies ‐ ISO: Inhibits Smad phosphorylation ‐ HDAC5 inhibitors: Restore Smad7 ‐ TSA, SDPR, TWEAK/Fn14 modulators ‐ ITGB1 antagonists (e.g., crizotinib)	[63, 64, 65, 66, 67, 68, 69, 70, 71, 72, 73, 74]
**Integrin–FAK**	‐ Mediates mechanotransduction via integrin clustering and FAK Tyr397 phosphorylation. ‐ Activates PI3K/Akt, ERK, mTOR; reinforces TGF‐β signaling. ‐ Modulated by ECM proteins (lumican) and EMT/EndMT transcription factors (Snail‐1).	Scar‐derived fibroblasts, endothelial cells, mouse wound models	‐ FAK inhibitors: Suppress fibrosis‐related genes ‐ Lumican: Inhibits fibroblast proliferation/ECM production ‐ Snail‐1 inhibition: Reduces EndMT and matrix remodeling	[75, 76, 77, 78, 79, 80, 81, 82, 83, 84]
**YAP/TAZ**	‐ Activated by ECM stiffness via integrin–FAK–RhoA–ROCK axis. ‐ Nuclear translocation promotes profibrotic genes (e.g., CTGF, COL1A1). ‐ ECM stiffness inhibits Hippo → YAP/TAZ translocation → fibrosis; RAP2 activates LATS1/2 → Hippo reactivation ‐ Crosstalk with TGF‐β and Wnt/β‐catenin pathways.	HSF cultures, Stretched fibroblasts; Rap2 overexpression models	‐ YAP/TAZ inhibitors: Block nuclear localization ‐ RAP2 activators: Restore Hippo pathway ‐ LATS1/2 agonists: Enhance Hippo signaling	[85, 86, 87, 88]
**PI3K/Akt**	‐ Activated by mechanical stimuli (e.g., micronegative pressure). ‐ Promotes fibroblast proliferation, angiogenesis, ECM production via mTOR. ‐ Interacts with TRP‐mediated calcium influx.	HaCaT cells, HSFs under negative pressure culture	‐ PI3K/Akt inhibitors: Attenuate fibrosis ‐ TRP channel blockers: Indirect inhibition via calcium influx	[89, 90, 91, 92]
**Rho GTPase**	‐ RhoA/ROCK promotes cytoskeletal remodeling and matrix contractility. ‐ Highly expressed in fibrotic tissue under stress. ‐ Inhibition reduces scar contracture and thickness.	In vitro fibroblast tension models, ROCK inhibitor studies	‐ ROCK inhibitors: Reduce fibrosis and improve wound remodeling	[93, 94]
**Ion Channels (TRP)**	‐ TRP‐mediated calcium influx regulates cytoskeleton and fibrosis. ‐ Mechanical stress increases TRP activity and downstream PI3K/Akt activation. ‐ Blocking Ca^2^⁺ entry reduces myofibroblast contractility.	In vitro fibroblast stretch models	‐ TRP channel blockers: Attenuate calcium‐mediated ECM deposition and cell tension	[91, 95]

#### 3.2.2 Developmental and Nonmechanical Pathways

In addition to the mechanical cues that contribute to fibroblast activation and matrix remodeling, a group of canonical developmental pathways—originally orchestrating embryonic patterning and stem cell fate—are aberrantly reactivated during wound healing and fibrosis. These signaling axes, including the Wnt/β‐catenin, Notch, Hedgehog, JAK/STAT, and MAPK/ERK axes, regulate diverse cellular behaviors, such as proliferation, migration, lineage commitment, and EMT, and their dysregulation underlies many pathological features of HTS and keloids. Non‐mechanical signaling pathways related to scar formation are listed in Table 2.

##### 3.2.2.1 Wnt/β‐Catenin Signaling

The Wnt/β‐catenin signaling pathway is a highly conserved regulatory axis that governs cell fate decisions, tissue homeostasis, and regeneration. Under normal physiological conditions, β‐catenin is tightly regulated by a cytoplasmic destruction complex composed of AXIN, adenomatous polyposis coli (APC), glycogen synthase kinase‐3 (GSK‐3), casein kinase 1 (CK1), and protein phosphatase 2A (PP2A) [96]. In the absence of Wnt ligands, this complex facilitates β‐catenin phosphorylation, leading to its ubiquitination and proteasomal degradation. This ensures minimal cytoplasmic accumulation and prevents inappropriate transcriptional activity [97].

Upon Wnt ligand binding to Frizzled (FZD) and low‐density lipoprotein receptor‐related protein 5/6 (LRP5/6), the destruction complex is disrupted, resulting in β‐catenin stabilization and cytoplasmic accumulation. Stabilized β‐catenin translocates into the nucleus, where it forms complexes with T‐cell factor/lymphoid enhancer factor (TCF/LEF) transcription factors, subsequently activating downstream genes involved in cell cycle progression (e.g., cyclin D1, c‐myc), tissue remodeling (FGF20), and negative feedback (DKK1) [98]. This canonical pathway not only drives proliferation and migration but also plays a critical role in ECM synthesis by modulating fibroblast behavior [99].

In the context of wound healing, transient Wnt/β‐catenin activation supports tissue regeneration by promoting stem cell self‐renewal, fibroblast activation, and granulation tissue formation [100]. However, sustained or dysregulated activation of Wnt/β‐catenin, as observed in HTS and keloids, leads to pathological fibrosis. Studies have shown that persistent β‐catenin activation in dermal fibroblasts enhances their proliferation, increases their motility, and elevates collagen Types I and III production. In animal models, forced β‐catenin stabilization exacerbates HTS formation, whereas β‐catenin inhibition alleviates fibrosis. Interestingly, β‐catenin deficiency in macrophages impairs the ability of these immune cells to migrate and adhere, potentially disrupting wound resolution and contributing to aberrant scar formation.

Wnt/β‐catenin signaling plays a pivotal role in HTS formation, and its inhibition has been shown to suppress fibroblast activation and ECM accumulation [101, 102], highlighting Wnt/β‐catenin as a viable antifibrotic target in preclinical models of scar modulation.

##### 3.2.2.2 Notch Pathway

The Notch pathway mediates direct cell–cell communication and regulates cell fate determination in skin repair. During wound healing, Notch signaling is transiently activated to guide keratinocyte differentiation and dermal remodeling. However, in HTS and keloids, Notch appears to be persistently upregulated, where it facilitates fibroblast activation, promotes EMT, and supports ECM overproduction [103]. Experimental suppression of Notch components such as Notch1 or its downstream effector Hes1 reduces fibroblast contractility and collagen Type I synthesis [104], indicating that the Notch pathway plays a fibrogenic role in skin scarring. The context‐dependent behavior of the Notch pathway—balancing regeneration and fibrosis—suggests that selective modulation rather than complete inhibition may offer therapeutic benefit.

##### 3.2.2.3 Hedgehog Signaling

The Hedgehog (Hh)‐GLI1 axis, a key regulator of developmental morphogenesis and adult tissue polarity, has recently been shown to play a central role in the maintenance of stem‐like keloid fibroblasts. Studies using patient‐derived keloid cells and ex vivo models have revealed the upregulation of Hh ligands and GLI1 transcriptional activity, which correlates with elevated expression of fibrosis‐related cytokines such as osteopontin. Notably, inhibition of Hh signaling via vismodegib attenuates ECM accumulation and reduces keloid tumor size in animal models, validating Hh as a viable antifibrotic target [105].

##### 3.2.2.4 JAK/STAT Axis

The JAK/STAT axis integrates cytokine signals with transcriptional programs involved in immunity, cell survival, and chronic inflammation. In keloid tissues, persistent activation of the JAK2/STAT3 cascade is associated with increased fibroblast proliferation, increased collagen production, and sustained M2 macrophage recruitment, creating a profibrotic microenvironment. Targeting this pathway via small‐molecule inhibitors (e.g., ruxolitinib) or siRNA‐mediated STAT3 silencing has shown promise in preclinical studies. A novel approach combining siBACH1‐based gene therapy with light‐activated delivery systems has been shown to simultaneously modulate the JAK/STAT and MAPK pathways, offering spatially controlled inhibition of keloid fibroblast activity [106].

##### 3.2.2.5 MAPK/ERK Pathway

The MAPK/ERK signaling cascade orchestrates key cellular processes, including proliferation, migration, and ECM remodeling. In both HTS and keloids, this pathway operates synergistically with TGF‐β to amplify fibrotic responses. Transcriptomic profiling of keloid fibroblasts revealed that the increase in ERK activity is mediated through the hypoxia‐inducible factor‐1α (HIF‐1α)/HOXC6 axis, suggesting that hypoxic stress in scar tissues may act as a driver of ERK‐dependent collagen synthesis and fibroblast proliferation [107]. These findings suggest that ERK inhibition is a rational antifibrotic strategy, especially in hypoxia‐enriched scar microenvironments.

**TABLE 2:** Nonmechanical signaling pathways implicated in HTS and keloid formation: recent advances in molecular mechanisms, research models, and therapeutic targets.

Pathway	Key findings	Research models	Potential therapeutic targets	References
**Wnt/β‐catenin**	Persistent β‐catenin activation promotes fibroblast proliferation, motility, and ECM overproduction; inhibition reduces fibrosis.	Human keloid fibroblasts; murine wound model	Wnt1, Wnt3a, β‐catenin, Porcupine, DKK1	[96, 97, 98, 99, 100, 101, 102]
**Notch**	Chronic Notch signaling promotes fibroblast activation, EMT, and collagen I production; inhibition of Notch1/Hes1 reduces fibrosis.	Human scar fibroblasts; 3D dermal models	Notch1, Hes1	[103, 104]
**Hh**	Hh‐GLI1 axis maintains keloid fibroblast stemness; inhibition with vismodegib reduces ECM accumulation and keloid mass.	Patient‐derived keloid cells; animal models	SHH, SMO, GLI1	[105]
**JAK/STAT**	JAK2/STAT3 pathway drives fibroblast proliferation and M2 macrophage recruitment; targeted therapies (e.g., ruxolitinib, siBACH1) show antifibrotic potential.	Keloid tissues; siRNA and light‐triggered systems	JAK2, STAT3, BACH1	[106]
**MAPK/ERK**	ERK signaling activated via HIF‐1α/HOXC6 under hypoxia promotes collagen synthesis; ERK inhibition suppresses fibrosis.	Keloid fibroblasts; hypoxic culture systems	ERK1/2, HIF‐1α, HOXC6	[107]

#### 3.2.3 Pathway Crosstalk and Emerging Targets

##### 3.2.3.1 Interactions Between TGF‐β and Wnt/β‐Catenin

Recent studies have increasingly highlighted that the pathogenesis of HTS and keloids arises not from isolated signaling events but from the complex interplay of multiple interconnected pathways. Among these, both mechanical and nonmechanical stress–responsive pathways—particularly the TGF‐β and Wnt/β‐catenin axes—have emerged as central regulators. TGF‐β1 is strongly activated by ECM stiffness and cellular contractility, whereas Wnt/β‐catenin signaling is frequently observed in anatomically high‐tension regions prone to keloid formation. Stabilized β‐catenin upregulates TGF‐β1 expression, while TGF‐β1, in turn, enhances β‐catenin nuclear translocation, creating a positive feedback loop that sustains fibroblast activation and excessive ECM deposition. Rather than functioning independently, these pathways exhibit extensive crosstalk and converge on shared downstream effectors and transcriptional programs that promote fibroblast activation, myofibroblast differentiation, and matrix overproduction. This reciprocal interaction reinforces a self‐perpetuating fibrotic loop, underscoring the need for integrated therapeutic strategies that target both axes simultaneously.

Moreover, β‐catenin interacts with Smad transcription factors, the principal mediators of TGF‐β signaling, to increase the transcription of fibrotic genes, including COL1A1, COL3A1, and α‐SMA. Additionally, Wnt signaling inhibits Smad7, a negative regulator of TGF‐β signaling, further potentiating TGF‐β‐induced fibrosis [108]. This cross‐talk provides a mechanistic basis for the synergistic effects of the Wnt/β‐catenin and TGF‐β pathways in HTS formation. Consequently, targeting the intersection of these pathways represents a potential strategy for attenuating excessive fibrosis while preserving regenerative functions [1].

Several recent studies have elucidated key molecular nodes at the interface of TGF‐β and Wnt signaling. A landmark investigation by Cohen et al. demonstrated that in idiopathic pulmonary fibrosis (IPF), TGF‐β1 activates fibroblasts to secrete secreted frizzled‐related protein 2 (sFRP2), a noncanonical Wnt ligand, which in turn drives epithelial metaplasia through Frizzled 5 (Fzd5)‐mediated activation of nuclear NFATc3 [109]. This work revealed not only a fibroblast‐dependent TGF‐β1/sFRP2‐Wnt signaling axis but also epigallocatechin gallate (EGCG) as a potential upstream inhibitor of both pathways by targeting TGF‐β1 signaling. Although this mechanism has been revealed in lung fibrosis, it may be highly relevant to dermal scarring, where EMT and fibroblast–keratinocyte crosstalk are similarly dysregulated. Another study by Hu et al. provided compelling mechanistic insight into how tartrate‐resistant acid phosphatase 5 (ACP5) acts as a bridge between TGF‐β and β‐catenin signaling. In a murine model of fibrosis, TGF‐β1 upregulated ACP5 expression via Smad3, which in turn dephosphorylated β‐catenin at Ser33/Thr41, preventing its degradation and enhancing its nuclear signaling activity [110]. This not only promoted fibroblast differentiation and proliferation but also positioned ACP5 as a dual‐pathway effector. Targeting ACP5 with small interfering RNA (siRNA) or small‐molecule inhibitors successfully reversed established fibrosis in vivo, indicating a promising strategy for addressing fibrotic conditions driven by intersecting TGF‐β and Wnt activity. In addition to canonical β‐catenin activation, the role of auxiliary regulators such as fatty acid‐binding protein 5 (FABP5) has also been revealed. In a PH‐LHD model, Lei et al. reported that FABP5 amplifies TGF‐β1‐induced fibroblast activation via GSK3β inhibition, thereby stabilizing β‐catenin [111]. Interestingly, the Wnt agonist SKL2001 abrogated the antifibrotic effect of FABP5 knockdown, again underscoring the bidirectional amplification loop between TGF‐β1 and Wnt signaling.

Collectively, these findings underscore a paradigm shift: rather than investigating these pathways in isolation, there is increasing impetus to identify convergent regulatory nodes that serve as shared therapeutic targets, offering another level of regulation for future interventions.

##### 3.2.3.2 Crosstalk With Novel Signaling Networks

The signaling cascades underlying fibrotic progression are complicated rather than solely involved in the interaction between TGF‐β and Wnt/β‐catenin. Recent evidence suggests that classical TGF‐β‐Smad signaling exerts its profibrotic influence through extensive crosstalk with other mechanotransductive and intracellular signaling pathways, including the PI3K/Akt, YAP/TAZ, and RhoA/ROCK pathways. These signaling modules not only respond to mechanical stimuli and ECM alterations but also serve as coregulatory elements that converge with TGF‐β at multiple transcriptional and posttranslational levels. For example, PI3K/Akt signaling has been shown to increase Smad3 phosphorylation, thereby potentiating the TGF‐β‐driven transcription of collagen and α‐SMA. In a recent study by Huang et al., the upregulation of Zyxin, a focal adhesion protein, was identified as a driver of skin fibrosis through integrin‐mediated coactivation of the FAK/PI3K/Akt and TGF‐β pathways, and genetic or pharmacologic inhibition of Zyxin attenuated fibrosis in both human keloid explants and murine models [112]. These findings underscore the functional integration of adhesion‐dependent and canonical profibrotic cues. Similarly, the YAP/TAZ transcriptional coactivators—regulated by cell density and matrix stiffness—can physically interact with Smad complexes to synergistically upregulate downstream effectors such as CTGF, a central mediator of hypertrophic scarring and keloid formation. These observations suggest that YAP/TAZ serve as molecular bridges linking mechanical stress to TGF‐β‐induced fibrogenesis. Indeed, in the fibrotic dermis, YAP nuclear localization is correlated with increased myofibroblast activity and matrix deposition. Emerging studies have further delineated the role of RhoA/ROCK signaling, which modulates cytoskeletal dynamics and cellular contractility, in facilitating TGF‐β‐mediated Smad activation and fibroblast‐to‐myofibroblast transition. This axis is particularly enriched in high‐tension anatomic regions that are prone to hypertrophic scarring, highlighting the regional specificity of signal integration.

In addition to TGF‐β signaling, emerging evidence suggests that YAP/TAZ functionally interact with the Trps1 and Wnt signaling pathways to regulate fibroblast fate. Multiomics analyses revealed that YAP/TAZ activation enhances Trps1 expression, which in turn negatively regulates Wnt signaling, suppresses fibroblast hyperactivation and promotes regenerative, nonscarring wound healing. Additionally, YAP/TAZ can directly modulate Wnt/β‐catenin signaling by interacting with β‐catenin and TEAD, forming a transcriptional complex that drives fibrotic gene expression. Fibroblast‐specific YAP knockout or pharmacological inhibition of YAP has been shown to disrupt Wnt‐mediated fibroblast activation, thereby reducing hypertrophic scarring and promoting scarless wound repair [113].

Collectively, these findings highlight the interconnected signaling architecture that underlies fibrotic progression. By identifying shared nodes, including Zyxin and YAP, future antifibrotic strategies may exploit multipathway inhibition to disrupt the synergistic signaling networks sustaining keloid and HTS formation.

##### 3.2.3.3 High‐Throughput Omics in Pathway Discovery

Advances in high‐throughput omics technologies—including scRNA‐seq, spatial transcriptomics, and quantitative proteomics—have deepened our understanding of HTS and keloids by revealing novel fibrotic signaling networks and cell‐type–specific regulatory changes. These tools provide high‐resolution analysis of transcriptional and proteomic landscapes, enabling the identification of dysregulated pathways such as the TGF‐β pathway and the PI3K/AKT/mTOR pathway in scar fibroblasts. In contrast to previous assumptions of fibroblast‐centric pathology, omics studies have revealed contributions from inflammatory and stromal cell populations.

Recent integrative analyses have delineated distinct fibroblast subtypes with specialized functions during scar progression. Spatial and lineage trajectory mapping revealed that POSTN⁺ mesenchymal fibroblasts—enriched in keloids—had increased TGF‐β signaling and ECM output, whereas IGFBP2⁺ fibroblasts in healthy skin presented antifibrotic phenotypes and resistance to TGF‐β–induced activation [114]. Retinoic acid was shown to reprogram pro‐inflammatory RBP5⁺ fibroblasts toward a matrix‐producing phenotype, thereby modulating collagen synthesis [115]. scRNA‐seq also revealed that TEM1 (CD248), a transmembrane protein enriched in scar fibroblasts, enhances TGF‐β signaling via receptor stabilization; its knockdown reduces ECM production in vivo, and therapeutic blockade via ontuxizumab has been shown to be effective in suppressing keloid growth [116].

Quantitative proteomics further highlighted the importance of posttranslational modifications in scar pathobiology. For example, S‐nitrosylated DJ‐1 was found to activate PI3K/AKT/mTOR signaling by inhibiting PTEN, promoting fibroblast proliferation and scar tissue formation; its pharmacological inhibition alleviated fibrosis in preclinical models. In addition, the fibroblast‐specific molecule NTM was identified through Mendelian randomization and single‐cell validation as a keloid‐associated regulator with therapeutic potential [117].

Collectively, these findings underscore the utility of single‐cell and spatial omics in revealing fibroblast heterogeneity and pathway‐specific vulnerabilities in pathological scarring, laying a foundation for precision therapeutic strategies.

### 3.3 Inflammatory and Immune Drivers of Fibrosis

The immune system plays another central role in regulating the pathological progression of HTS and keloids through immune cell infiltration, cytokine secretion, and intracellular signaling cascades. Unlike normal wound healing, where inflammation resolves in a timely manner, HTS and keloids are sustained by chronic immune activation, leading to a prolonged profibrotic microenvironment. This persistent inflammatory response not only promotes excessive collagen synthesis but also prevents proper tissue remodeling, resulting in disorganized ECM architecture and scar hypertrophy. Understanding the immunological mechanisms underlying HTS and keloid formation is crucial for developing targeted interventions that modulate inflammatory and fibrotic pathways to prevent or reduce pathological scarring.

#### 3.3.1 Immune Cell Contributions

Macrophages are pivotal regulators of wound healing and are capable of adopting distinct phenotypes according to microenvironmental signals [118]. Pro‐inflammatory M1 macrophages secrete cytokines such as TNF‐α and interleukin‐6 (IL‐6), initiating immune responses and microbial clearance [119]. In contrast, anti‐inflammatory M2 macrophages release TGF‐β1 and interleukin‐10 (IL‐10), promoting fibroblast activation, myofibroblast differentiation, and ECM deposition. Studies have demonstrated that M2 macrophages are predominant in HTS and keloids, where they sustain fibrosis and tissue remodeling [120]. Macrophage depletion models show significantly reduced scar formation, confirming their fibrogenic role. Additionally, elevated levels of pro‐inflammatory cytokines such as IL‐1α, IL‐1β, IL‐6, and TNF‐α in keloids contribute to chronic inflammation by increasing fibroblast proliferation, inhibiting apoptosis, and driving ECM synthesis. Interestingly, while generally considered anti‐inflammatory, IL‐10 plays paradoxical roles in pathological scarring by attenuating fibrosis through the suppression of inflammation or facilitating fibrogenesis through M2 macrophage polarization and fibroblast activation, underscoring the need for context‐dependent therapeutic approaches.

Mast cells also play important roles in pathological scarring and are found at increased densities in HTS and keloids. Upon activation, they release histamine, tryptase, and chymase, which stimulate fibroblast proliferation, collagen synthesis, and ECM remodeling [118]. Moreover, mast cell mediators promote angiogenesis, supporting the metabolic demands of expanding fibroblast populations [121]. Therapeutic strategies using histamine antagonists and mast cell stabilizers have been explored to mitigate fibrosis in keloid‐prone individuals.

Lymphocytes, particularly adaptive immune cells, contribute to fibrosis through cytokine secretion and immune regulation [122]. T helper 2 (Th2) cells produce IL‐4, IL‐5, and IL‐13, which increase fibroblast proliferation, collagen synthesis, and ECM deposition [123]. Elevated IL‐4 and IL‐13 levels in keloids correlate with increased fibrosis severity. Regulatory T cells (Tregs) further support the fibrotic microenvironment by secreting TGF‐β1, perpetuating fibroblast activation and matrix accumulation. Conversely, CD4⁺ T cells have also been shown to exert antifibrotic effects under certain conditions, as their presence can suppress inflammation and limit collagen deposition, thereby reducing scar formation [124]. These findings highlight that lymphocyte subpopulations play divergent and sometimes opposing roles in fibrosis, suggesting that immune‐mediated regulation of scar formation is a highly complex and context‐dependent process. The targeting of Th2 cytokine signaling is being investigated as a strategy to control pathological scarring.

#### 3.3.2 Inflammatory and Immune Mediators

In addition to immune cells, a variety of cytokines, chemokines, and growth factors maintain chronic inflammation and fibrosis in HTS and keloids. Chemokines such as CCL2 (monocyte chemoattractant protein‐1) and CXCL8 (IL‐8) are highly expressed by keloid fibroblasts and keratinocytes and promote immune cell recruitment and sustained inflammation [125]. CCL2 specifically enhances macrophage infiltration, whereas CXCL8 facilitates neutrophil accumulation, exacerbating tissue damage via oxidative stress and protease release.

Key growth factors—including vascular endothelial growth factor (VEGF), platelet‐derived growth factor (PDGF), and connective tissue growth factor (CTGF)—promote angiogenesis, fibroblast proliferation, and ECM remodeling [126, 127]. VEGF‐driven capillary formation supports fibroblast metabolic demands and accelerates collagen deposition. Pharmacological inhibition of VEGF and other profibrotic factors represents a promising therapeutic avenue.

At the intracellular signaling level, the TGF‐β/Smad pathway is central to fibrosis. TGF‐β1 secreted by immune cells and fibroblasts activates Smad2/3, driving fibroblast proliferation and ECM accumulation [128]. Persistent activation prevents normal wound resolution, leading to scar hypertrophy. Additionally, inflammasomes—especially NLRP3—in macrophages and fibroblasts activate the caspase‐1‐mediated secretion of IL‐1β and IL‐18, amplifying inflammation and promoting the fibroblast‐to‐myofibroblast transition [129]. Targeting these pathways, including TGF‐β receptors, Smad proteins, and the NLRP3 inflammasome, as well as modulating immune checkpoints to suppress profibrotic immune responses, are under active investigation to mitigate pathological scarring.

## 4 Therapeutic and Preventive Interventions

Given the multifactorial and heterogeneous nature of HTS and keloids, clinical management remains a significant challenge. Despite *significant* advances in understanding shared and lesion‐specific fibrotic pathways, no single treatment has proven universally effective. Current therapeutic strategies encompass *options*—from physical ablation and pharmacologic modulation to emerging molecular and biomechanical interventions—each tailored to the lesion's behavior, location, and patient‐specific characteristics.

Optimal management therefore requires an individualized, *mechanism‐based* approach that addresses both the biological complexity and clinical variability of pathological scars.

The following sections review *recent advances* in invasive interventions, noninvasive therapies, and novel emerging strategies *aimed at* improving scar prevention and treatment outcomes.

### 4.1 Invasive Interventions

#### 4.1.1 Surgical Excision

**FIGURE 3:**
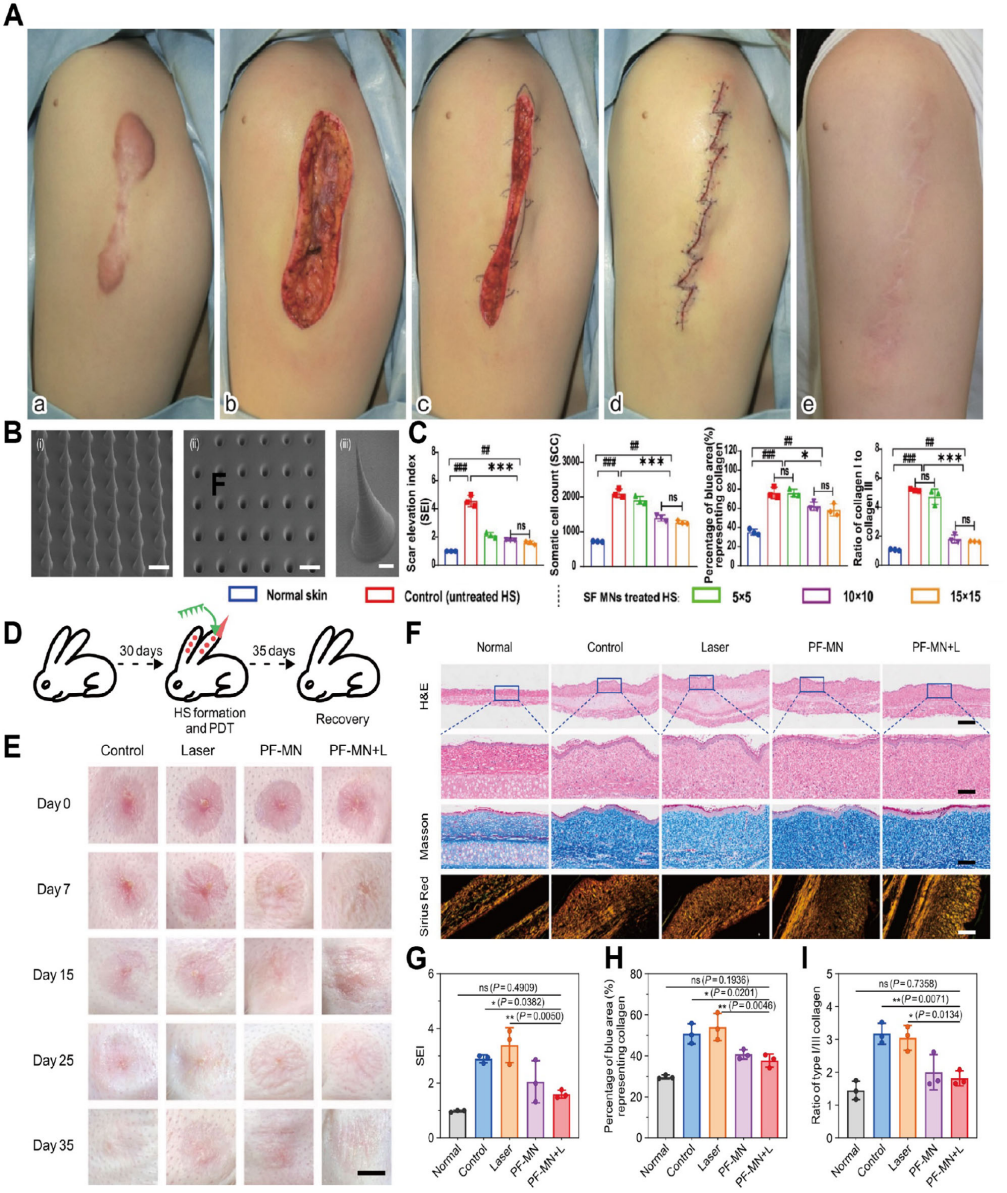
Illustrative examples of new invasive therapeutic interventions (A) immediately after surgery. (e) Two years after surgery. Keloids developed from BCG vaccination. It was excised, tension was released by fascial sutures, and z‐plasties were performed. Postoperative radiotherapy (18 Gy/3 fr/3 days) was then administered. No recurrence was observed 2 years after surgery [22, 130]. (B) SEM images showing the (i) side and (ii) bottom views of the shell of the PF‐MN patch. Scale bar, 500 µm. (iii) Enlarged SEM image displaying one PF‐MN. Scale bar, 100 µm [141]. (C) Quantification of the SEI, cellularity, and collagen content and the ratio of Type I to Type III collagen from the images in B (^###^ *p*<0.001, ^##^ *p*<0.01, and ^#^ *p*<0.05 compared with normal skin and ^***^ *p*<0.001, ^**^ *p*<0.01, and ^*^ *p*<0.05 compared with the control group, *n* = 3) [137]. (D) Experimental schematic of PF‐MNs for the PDT of HS [141]. (E) Photographs of scar tissue from the ears of rabbits after various treatments at different times. Scale bar, 5 mm [141]. (F) Representative H&E staining, Masson trichrome staining, and Sirius Red staining images of HS on Day 15. Scale bar, 1 mm for H&E‐stained images (top), 200 µm for H&E‐stained images (enlarged), Masson's trichrome‐stained images, and Sirius Red‐stained images [141]. (G–I) Quantitative analysis of the SEI (G), percentage of collagen (H), and Type I/III collagen ratio (I) of HS (*n* = 3 biologically independent samples). bPF‐MN: blank PF‐MN. Three independent experiments were performed, and representative results are shown in I. Data are presented as the means ± SDs, and statistical significance was analyzed via one‐way ANOVA with Tukey's multiple comparison test. *Source*: Ogawa et al. [130] and Zhang et al. [141].

Surgical excision remains a cornerstone treatment for HTS and keloids, although its effectiveness as a standalone therapy is limited by high recurrence rates—up to 45%–100% for keloids following excision alone. In HTS, surgery typically involves the excision of thickened, hyperpigmented, and tensioned scar regions, followed by tension‐free closure to promote normal healing. For larger or high‐tension defects, reconstructive techniques such as Z‐plasty, W‐plasty, local flap transfers, or full‐thickness skin grafts redistribute mechanical stress, minimizing contracture risk and improving functional and cosmetic outcomes (Figure 3A). Case series studies have shown that Z‐plasty combined with radiotherapy significantly reduces anterior chest and upper‐arm keloid recurrence rates to 10.6% and 5.3%, respectively [22, 130]. Mechanistically, Z‐plasty alters the direction and distribution of mechanical tension by redistributing stress vectors away from the original wound axis. This mechanical offloading may indirectly attenuate mechanosensitive fibrogenic signaling—particularly TGF‐β1 expression—thereby reducing fibroblast activation and ECM deposition [131]. Given the strong correlation between mechanical tension and scar recurrence, tension‐reducing suturing, especially at the subcutaneous fascia level, plays a vital role in lowering dermal tension and recurrence rates. In keloid management, smaller lesions may be radically excised, whereas larger or multiple lesions often require partial or core excision to remove fibrotic centers while preserving surrounding tissue, thereby reducing postoperative fibroproliferation and mechanical stress.

Recent surgical advancements emphasize mechanical tension minimization to improve outcomes. Ultrareduced tension sutures and optimized incision designs enhance skin approximation, decrease horizontal tension, and foster healthier dermal remodeling. Customized surgical planning tailored to scar morphology, location, and size enables more precise tension redistribution. Emerging fascial remodeling surgery represents a promising innovation in this area. By suturing superficial and/or deep fascia layers, mechanical tension is further reduced, blocking muscle‐derived forces and limiting fibroblast activation at a key mechanotransductive level. Clinical data show that combining fascia tensile reduction with excision and radiotherapy can dramatically decrease recurrence rates—from approximately 43% with traditional methods to approximately 5.2% in severe anterior‐chest keloids—while enabling primary closure of large defects without grafts or flaps, reducing donor site morbidity [132, 133]. These fascia‐guided repairs capitalize on recent insights into fascia fibroblast migration and ECM dynamics [1, 134], disrupting the fibrotic feedback loops that underlie HTS and keloid formation.

Owing to high recurrence rates after surgery alone, adjuvant therapies are integral to scar management. Postoperative radiation therapy, including electron beam and high‐dose‐rate superficial brachytherapy, administered within 24 h postexcision, reduces keloid recurrence to less than 10%, with a favorable long‐term safety profile. Intralesional corticosteroids, particularly triamcinolone acetonide (TAC), which is often combined with agents such as 5‐FU or verapamil, further suppress fibroblast proliferation and collagen deposition, lowering recurrence rates to 7.5%–23.3%. Emerging modalities such as fractional CO_2_ laser‐assisted drug delivery (LADD) and cryotherapy show promise in augmenting scar remodeling and volume reduction, especially when integrated with surgery and steroid injections. Silicone gel sheets, pressure therapy, and other biomechanical interventions remain valuable adjuncts within comprehensive, multimodal treatment protocols. Overall, the combination of refined surgical techniques and evidence‐based adjuvant therapies enables more durable scar control and functional restoration.

#### 4.1.2 Microneedling Approaches

Microneedling (MN) technology has emerged as a promising approach for targeting HTS, offering minimally invasive, drug‐delivery capabilities with high biocompatibility. Recent advancements include bioloaded MN patches that integrate anti‐inflammatory and antifibrotic agents [135]. For example, photothermal soluble hyaluronic acid MN patches have been shown to reduce fibroblast proliferation and collagen deposition, effectively preventing HTS formation [136]. Silk fibroin MNs (SFMNs) reduce fibroblast contraction and local mechanical stress and downregulate TGF‐β1, α‐SMA, Type I collagen, and fibronectin expression, leading to a low‐pressure microenvironment that mitigates scar formation [137].

Bilayered MNs loaded with dexamethasone and colchicine (DC‐MNs) offer dual‐phase therapeutic release: the outer layer rapidly delivers dexamethasone to inhibit pro‐inflammatory macrophage polarization, whereas the inner layer gradually releases colchicine to suppress myofibroblast overproliferation and disorganized collagen deposition [135]. Additionally, dissolving MN arrays with a tip‐concentrated design has improved mechanical penetration and enhanced triamcinolone delivery, resulting in better therapeutic outcomes [138].

Endogenous stimulus‐responsive MNs further refine treatment precision by modulating fibroblast–keratinocyte crosstalk through the HSPG2–DAG1 ligand–receptor axis, revealing a novel mechanistic pathway with broad therapeutic potential [139]. Another innovation, the programmed functional MN array (PFMN), aligns treatment with dynamic wound‐healing phases: during early inflammation, laser‐activated reactive oxygen species (ROS) eliminate bacterial biofilms; in later stages, the ROS‐sensitive MN shell degrades, exposing a verteporfin‐loaded core that suppresses Engrailed‐1 activation in fibroblasts, thus preventing pathological scarring [140, 141]. These effects have been validated in both acute and chronic wound models, as shown in Figure 3B.

In addition to patch‐based technologies, percutaneous collagen induction (PCI), also known as surgical needling, uses 3‐mm needles to create controlled microinjury across the dermis without damaging the basal layer. This induces growth factor expression and intradermal bleeding, stimulating collagen remodeling through a regenerative wound‐healing cascade [142].

Together, these MN strategies—including drug‐loaded MNs, responsive release systems, and mechanical induction techniques—provide a multimodal, targeted approach to HTS management, effectively addressing both biological signaling and tissue mechanics.

#### 4.1.3 Intralesional Drug Therapies

##### 4.1.3.1 Corticosteroid Injections

Corticosteroids, particularly intralesional TAC, are widely regarded as first‐line treatments for HTS and keloids because of their ability to inhibit fibroblast proliferation by suppressing the MAPK and PI3K/Akt pathways, inducing apoptosis via caspase‐3 activation and Bcl‐2/Bax modulation, and attenuating TGF‐β1/Smad2/3 signaling. Additionally, they downregulate VEGF signaling to limit angiogenesis and excessive collagen deposition. TAC injection has response rates ranging from 50% to 100%, although the incidence of recurrence varies from 9% to 50%, depending on lesion characteristics and treatment protocol [143]. To reduce side effects such as skin atrophy, hypopigmentation, and telangiectasia, TAC is often combined with agents such as 5‐FU or laser therapy [144]. The Japanese guidelines now favor corticosteroid tapes or plasters as first‐line treatment, reserving injections for refractory lesions.

##### 4.1.3.2 5‐Fluorouracil Injection

5‐Fluorouracil (5‐FU), a chemotherapeutic agent, inhibits fibroblast proliferation by targeting thymidylate synthase and disrupting DNA replication while also downregulating TGF‐β1 via transcriptional inhibition [145]. When used alone or with TAC, 5‐FU reduces recurrence and mitigates corticosteroid‐related side effects. A randomized trial revealed lower rates of atrophy (8%) and telangiectasia (21%) with 5‐FU/TAC than with TAC monotherapy (44% and 50%, respectively), although no significant difference in remission was noted at six months [146]. Additionally, transdermal transfersome nanogels codelivering TAC and 5‐FU enhance antifibrotic efficacy by promoting M2 macrophage polarization via IL‐4/IL‐13 and reducing COL1A1/COL3A1 expression through TGF‐β1/Smad3 inhibition, suggesting dual anti‐inflammatory and antifibrotic effects [147].

Imiquimod, a TLR agonist, modulates immune activity by upregulating TNF‐α, IFN‐α, IL‐1, IL‐6, and IL‐8, which in turn activate STAT1/3 signaling to suppress fibroblast activity and enhance matrix degradation via MMPs. Despite its mechanistic promise, clinical outcomes remain inconsistent. A meta‐analysis of seven studies revealed a 39% recurrence rate after topical 5% imiquimod use, with limited efficacy in preventing recurrence postexcision, highlighting the need for further clinical validation.

#### 4.1.4 Laser‐Based Therapies

Laser therapy represents a cornerstone in the treatment of HTS and keloids, offering noninvasive remodeling of scar architecture. While early laser applications (e.g., argon and CO_2_ lasers in the 1980s) were limited in efficacy, modern developments—particularly fractional ablative and vascular‐selective lasers—have significantly improved clinical outcomes [148].

Fractional ablative lasers, such as CO_2_ and erbium:YAG lasers, deliver thermal microinjuries to dermal water‐containing tissues, promoting controlled fibrosis reduction and stimulating the formation of organized collagen fibrils [149]. Pulsed dye lasers (PDLs), operating at 585–595 nm, target oxyhemoglobin to ablate hypervascular components of scars. These lasers are especially effective for treating erythematous HTS, whereas fractional CO_2_ lasers show superior efficacy in treating thicker, hypopigmented lesions [150, 151, 152].

LADD has emerged as a critical mechanism underlying the increased efficacy of fractional laser therapy in HTS and keloids. By generating controlled microablative channels within the epidermis and superficial dermis, fractional CO_2_ lasers significantly improve the transdermal penetration of topical agents such as TAC and betamethasone, thereby augmenting therapeutic outcomes in terms of scar texture, hypertrophy, and pigmentation normalization [153]. This mechanistic insight aligns with recent clinical evidence demonstrating the superiority of combination laser therapy over monotherapy: a systematic review revealed that integrating lasers with corticosteroids or 5‐FU yielded greater improvements in patient‐reported pain scores (0.580 vs. 0.420), more than 50% enhancement in scar appearance, and reduced recurrence rates (18.7% vs. 24.9%) [154]. While not always explicitly labeled LADD, many of these combination regimens functionally rely on laser‐facilitated drug penetration to achieve synergistic effects. Ongoing studies—ranging from wavelength‐specific clinical trials to preclinical modeling—aim to refine laser parameters and optimize drug‒laser pairings, underscoring LADD's potential as a platform for personalized, phenotype‐targeted therapy. As such, LADD represents a pivotal advancement in scar management, enabling integrated functional and esthetic improvement in both HTS and keloids.

Emerging research continues to refine laser‐based strategies. For example, comparative trials and preclinical models are evaluating wavelength‐specific responses and optimized laser‒drug combinations, offering insights into tailored therapy for various scar phenotypes. These advances highlight the potential of integrated laser systems in achieving both functional and esthetic improvements in HTS and keloid management. Key invasive therapies for HTS and keloids are summarized in Table 3.

**TABLE 3:** Invasive therapies for HTS and keloids: mechanisms, pathways, and evidence from preclinical and clinical studies.

Therapy type	Mechanism of action	Key targets/pathways	Research models	Therapeutic potential	Clinical trial information	References
**Surgical excision**	‐ Removal of fibrotic tissue. ‐ Redistribution of mechanical tension via Z‐/W‐plasty, grafts, or fascia remodeling. ‐ Tension‐free closure lowers recurrence by reducing dermal tension and fibroblast activation. ‐ Fascia suturing minimizes muscle‐derived forces and disrupts fibrotic feedback loops.	Mechanical stress, TGF‐β1, ECM signaling	Clinical cases, surgical techniques	‐ Recurrence as low as 5.2% with fascia remodeling. ‐ Improved functional/cosmetic outcomes. ‐ Essential for reducing mechanical drivers of recurrence.	NCT02922972	[22, 130, 131, 132, 134]
**MN**	‐ Drug delivery via microneedle patches (e.g., SFMNs, PFMNs). ‐ Downregulates TGF‐β1, α‐SMA, COL1, FN1. ‐ Modulates fibroblast–keratinocyte crosstalk via HSPG2–DAG1 axis. ‐ PFMN enables wound‐phase–aligned drug release. ‐ PCI creates controlled dermal injury to trigger regenerative remodeling.	TGF‐β1, FN1, α‐SMA, HSPG2–DAG1, Engrailed‐1	Preclinical models, MN platforms	‐ Reduced fibroblast contraction and collagen deposition. ‐ Enhanced scar remodeling and vascular normalization. ‐ Improved precision of drug release and delivery.	MS‐65‐2022	[135, 136, 137, 138, 139, 140, 141, 142]
**Intralesional drugs**	‐ TAC: Suppresses MAPK, PI3K/Akt, and VEGF; induces apoptosis via caspase‐3 and Bcl‐2/Bax modulation. ‐ 5‐FU: Inhibits thymidylate synthase and TGF‐β1 transcription. ‐ TAC/5‐FU nanogels enhance M2 polarization and Smad3 inhibition. ‐ Imiquimod: Activates STAT1/3 via TLRs; promotes MMP‐mediated ECM degradation.	MAPK, PI3K/Akt, TGF‐β1/Smad, STAT1/3, VEGF	Clinical trials, in vitro fibroblast studies	‐ High efficacy with TAC/5‐FU combos (50%–100% response). ‐ Lower atrophy/telangiectasia with 5‐FU vs. TAC monotherapy. ‐ Variable efficacy with imiquimod (39% recurrence).	TCTR20220318004; IRCT20210524051384N6; NCT02155439	[143, 144, 145, 146, 147]
**Laser therapy**	‐ Ablative (CO_2_, erbium:YAG) and vascular‐selective (PDL) lasers remodel ECM and ablate hypervascularity. ‐ LADD enhances penetration of triamcinolone or 5‐FU via fractional CO_2_ microchannels. ‐ Combination therapy improves scar appearance, pain scores, and recurrence. ‐ Wavelength optimization under investigation for phenotype‐specific treatment.	ECM remodeling, vascular markers, drug penetration pathways	Clinical studies, preclinical scar models	‐ >50% improvement in scar appearance. ‐ Lower recurrence with LADD (18.7% vs. 24.9%). ‐ Enhanced drug efficacy and scar texture normalization.	NCT02155439; ChiCTR2400080148	[148, 149, 150, 151, 152, 153, 154]

### 4.2 Noninvasive Interventions

#### 4.2.1 Negative Pressure Wound Therapy

HTS and keloids are frequent sequelae of delayed or complicated wound healing. Chronic or suboptimally managed wounds often promote prolonged inflammation, hypoxia, and fibroblast dysregulation, all of which contribute to excessive ECM deposition and fibroproliferative scarring [155]. Persistent expression of profibrotic markers such as TGF‐β1, En1‐lineage fibroblast activation, and poor neovascularization further exacerbate scar hypertrophy and contracture.

**FIGURE 4:**
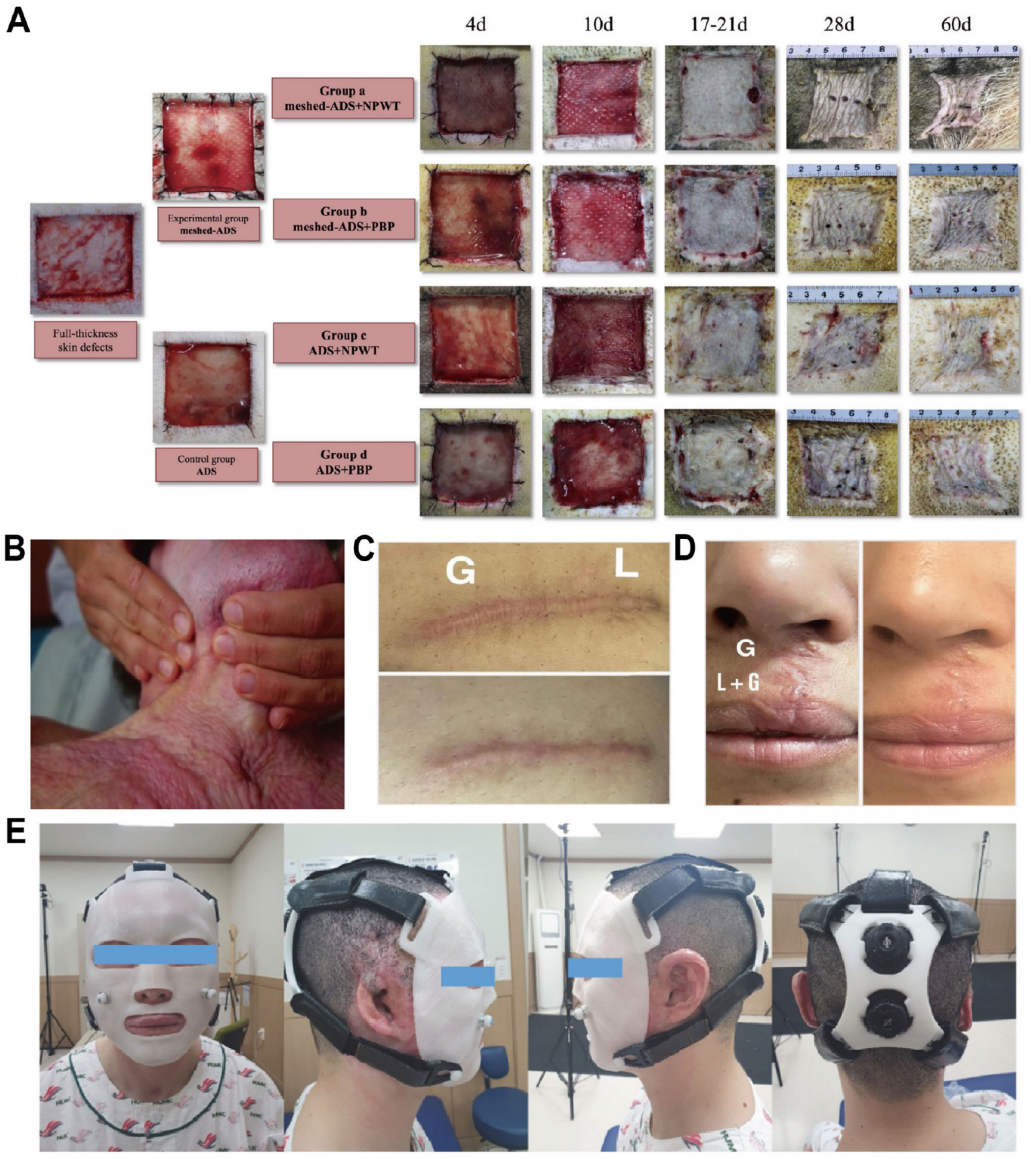
Illustrative examples of noninvasive therapeutic interventions. (A) General observations of the in vivo study. Dynamic progression of wound healing in the ADS + NPWT (Group a), ADS + PBP (Group b), ADS + NPWT (Group c), and ADS + PBP (Group d) groups at Day 4, Day 10, Days 17–21, Day 28, and Day 60 after the first‐stage operation [10]. Manual massages for the treatment of immature scars [173]. (B) Diagram of massage and manual therapy. (C) A 26‐year‐old female patient was sent with a postbenign breast mass removal surgery scar for 4 months; the upper image was taken before treatment, and the lower image was taken after treatment. The right side was treated with silicone gel and a 577‐nm diode laser, and the left side was treated with silicone gel only [173]. (D) A 42‐year‐old female patient presented with a cleft lip surgery scar for 1 month; the left image was pretreated, and the right image was posttreatment. The lower half was treated with silicone gel and a 577‐nm diode laser, and the upper half was treated with silicone gel only [182]. (E) Patient application of the 3D mask. The patients could correct pressure levels by adjusting the strap length of the mask's 5‐point harness system on their own [177]. *Source*: Wei et al. [156], Téot et al. [10], Hwang et al. [177].

NPWT, which involves the application of controlled subatmospheric pressure to the wound bed, has emerged as a frontline strategy to accelerate wound closure, prevent chronic inflammation, and reduce fibrotic remodeling. It maintains a moist microenvironment, removes exudates, improves perfusion, and delivers mechanical cues that stimulate tissue regeneration [10, 156], as shown in Figure 4A. Clinical and experimental data suggest that early intervention with NPWT reduces the likelihood of pathological scarring, particularly in high‐risk wounds such as burns, diabetic ulcers, and trauma [157].

Mechanistically, NPWT modulates the fibrotic cascade through multiple pathways. Studies indicate that NPWT enhances angiogenesis and re‐epithelialization by upregulating VEGF and basic fibroblast growth factor (bFGF) while downregulating inflammatory cytokines and TGF‐β1 [155]. Importantly, recent preclinical work has shown that NPWT activates the mechanosensitive transcription factor YAP, which promotes fibroblast proliferation but concurrently inhibits the persistence of En1‐lineage fibroblasts, a key driver of pathological scarring [158]. This dual regulation contributes to more balanced ECM remodeling and reduced scar thickness.

Advanced approaches have combined NPWT with biomaterial scaffolds to improve efficacy. For example, Wei et al. designed a layered mesh scaffold seeded with adipose‐derived stem cells (ADSCs), which, under NPWT, improved subcutaneous drainage, cell survival, and tissue integration, accelerating wound repair while limiting fibrotic overgrowth [159].

Clinically, NPWT has demonstrated tangible benefits in reducing scar hypertrophy. A long‐term follow‐up study revealed that postburn patients treated with NPWT had significantly flatter, less vascularized scars on the Vancouver scar scale than did those receiving standard care [160]. These outcomes were attributed to the early mechanical modulation of fibroblast activity and vascular maturation.

Collectively, these findings highlight NPWT not only as a wound healing facilitator but also as a promising antifibrotic therapy. Its ability to alter local mechanical and molecular signaling early in wound healing offers valuable therapeutic implications for minimizing HTS and keloid formation in both acute and chronic wounds.

#### 4.2.2 Manual and Mechanical Scar Modulation

Manual scar therapies aim to improve scar appearance and function by modulating local tissue mechanics, reducing inflammation, and promoting collagen remodeling [10], which is particularly valuable in managing HTS and keloids. Common manual modalities include massage, dry needling, fascial release, and kinesiotaping, each targeting distinct aspects of scar biology and biomechanics.

##### 4.2.2.1 Massage and Manual Therapy

Massage therapy is widely used to promote circulation, lymphatic drainage, and collagen realignment in scar tissue. Clinical trials have shown that regular massage reduces scar thickness and improves pliability, particularly when combined with other physical modalities [161, 162]. Deep‐tissue techniques may further relieve tension and aid in collagen remodeling, but excessive pressure can aggravate inflammation, making technique, timing, and pressure customization essential [163].

##### 4.2.2.2 Dry Needling and Fascial Release

Dry needling involves the insertion of fine needles into scar tissue to stimulate local microcirculation, degrade fibrotic adhesions, and promote collagen turnover. In clinical settings, this technique has led to significant reductions in scar height and improved texture, with one study reporting a 40% decrease in thickness after 6 weeks of treatment [164]. When combined with fascial release, it enhances mobility and reduces contracture, offering both structural and symptomatic relief in HTS and keloid management.

##### 4.2.2.3 Kinesiotaping Techniques

Kinesiotaping, a noninvasive technique, uses elastic tape over scarred areas to decrease mechanical tension, support soft tissue, and improve local perfusion. By gently lifting the skin, it facilitates lymphatic flow and reduces pressure on underlying structures. Clinical research has shown that kinesiotaping can lead to 20%–30% reductions in scar height and better elasticity, particularly in joints and high‐mobility areas [165].

##### 4.2.2.4 Combined Approaches: Synergy and Limitations

Multimodal manual therapy often yields synergistic outcomes, addressing multiple scar‐related dysfunctions simultaneously. Studies combining massage, dry needling, and kinesiotaping have reported greater improvements in pliability and symptom relief than single interventions do, with up to 35% of patients experiencing complete resolution of pruritus and pain [162]. Mechanistically, these therapies are thought to act through mechanotransduction, local immune modulation, and ECM remodeling. For example, massage may downregulate TGF‐β1 and IL‐6 and promote M2 macrophage polarization; dry needling may activate fibroblast apoptosis via mechanical signaling; and kinesiotaping may modulate cutaneous mechanoreceptors and reduce subclinical inflammation.

Despite promising results, manual scar therapies require individualized application to avoid complications such as exacerbated fibrosis, especially during early wound healing [163]. A major limitation remains the lack of standardized protocols regarding treatment frequency, intensity, and assessment metrics. Research should focus on establishing evidence‐based guidelines and identifying biomarkers to tailor interventions on the basis of each patient's wound status and fibrotic activity profile.

#### 4.2.3 Silicone‐Based Dressing

Silicone‐based dressings, including sheets and gels, are widely accepted as first‐line interventions for HTS and keloids because their occlusive, hydrating properties reduce transepidermal water loss (TEWL), modulate fibroblast activity, and inhibit excessive collagen synthesis  [166, 167]. Their mechanical barrier also stabilizes scar tissue and improves pliability, contributing to reduced thickness and pruritus  [168]. Comparative studies indicate that both silicone sheets and gels effectively lower the TEWL, although gels are often favored for their ease of use despite limitations such as drying time and surface sheen [169].

Combination approaches further enhance efficacy. Silicone sheets combined with intralesional corticosteroids have shown additive effects in reducing scar progression and inflammation  [170], whereas randomized trials have demonstrated the noninferiority of silicone gels compared with pressure garment therapy (PGT) for pediatric burns, suggesting greater comfort and compliance potential  [171]. Meta‐analyses confirmed the role of silicone in lowering recurrence rates, reinforcing its position as a clinical mainstay in scar management  [149, 172, 173] (Figure 4C,D).

Recent investigations have explored alternatives, such as medical moisturizers such as alhydran and DermaCress, which offer comparable hydration effects and longer‐lasting TEWL reduction at lower costs [174]. Although further validation is needed, these options may suit patients seeking more affordable or less restrictive treatments. Overall, silicone‐based therapies remain the benchmark for conservative scar care, with robust evidence supporting their efficacy in both prevention and long‐term control  [138].

#### 4.2.4 Pressure Garments and Skin Tapes

PGT is a widely used noninvasive method for managing HTS, particularly postburn HTS, by applying sustained mechanical pressure to modulate collagen remodeling and reduce scar thickness [175, 176]. Optimal therapeutic effects rely on maintaining consistent pressure levels, yet garment fit and patient adherence often limit efficacy [177]. Although commonly employed, the effectiveness of PGT remains controversial: a systematic review of 15 RCTs involving over 1100 patients reported inconsistent benefits regarding scar height, pruritus, and elasticity, likely due to heterogeneity in pressure magnitude, duration, and garment design  [178, 179, 180].

Despite limited standalone evidence, PGT is frequently combined with silicone‐based therapies or topical agents to improve outcomes [181]. Recent innovations, such as customized 3D‐printed garments integrated with pressure sensors, have improved treatment precision and patient comfort. A randomized trial using sensor‐enabled compression masks revealed significant reductions in scar thickness and improved hydration and pliability after 12 weeks  [177] (Figure 4E). These advances suggest that personalized, feedback‐guided pressure application may overcome prior limitations.

While PGT continues to be a cost‐effective option, its role should be guided by individual scar characteristics and patient tolerance. Further large‐scale studies are essential to define standardized protocols, clarify patient selection, and establish the optimal therapeutic pressure range. Main noninvasive therapies for HTS and keloids are summarized in Table 4.

**TABLE 4:** Noninvasive therapies for HTS and keloids: mechanisms, pathways, and evidence from preclinical and clinical studies.

Intervention	Mechanism of action	Therapeutic outcomes	Limitations	Clinical trial information	References
**NPWT**	Enhances angiogenesis and re‐epithelialization via VEGF/bFGF; reduces TGF‐β1 and inflammatory cytokines; activates YAP to modulate fibroblast lineage and ECM remodeling	Accelerated wound closure; reduced scar thickness; improved vascular maturation; prevention of pathological scarring	Requires early intervention; cost and equipment access may be limiting	NCT01480362; DRKS00003347;ACTRN12622000044729; ACTRN12616001100482	[10, 155, 156, 157, 158, 159, 160]
**Massage and manual therapy**	Promotes circulation, lymphatic flow, and collagen realignment; may downregulate TGF‐β1 and IL‐6; supports M2 macrophage polarization	Improved scar pliability and thickness; enhanced patient comfort and mobility	Risk of exacerbating inflammation if technique is inappropriate; requires personalization	NCT01131904	[161, 162, 163]
**Dry needling and fascial release**	Stimulates microcirculation; mechanically disrupts fibrotic adhesions; induces fibroblast turnover and apoptosis	Up to 40% scar thickness reduction; enhanced texture and contracture relief	Technique‐sensitive; optimal frequency and duration not well standardized	−	[164]
**Kinesiotaping**	Reduces skin tension; improves local perfusion and lymphatic flow; may modulate mechanoreceptors and decrease subclinical inflammation	20%–30% reduction in scar height; improved elasticity in high‐mobility areas	Limited by tape adherence and placement variability; more data needed on long‐term efficacy	−	[165]
**Combined manual therapies**	Synergistic effects via mechanotransduction, immune modulation, ECM remodeling (e.g., massage + needling + taping)	Up to 35% complete resolution of symptoms (e.g., pruritus, pain); superior pliability vs. monotherapies	Lack of standardized protocols; individualized treatment necessary	−	[162]
**Silicone‐based dressings**	Occlusion and hydration reduce TEWL; stabilize scar tissue; suppress collagen synthesis and fibroblast activity; Hydration and barrier restoration; alternatives to silicone (e.g., Alhydran, DermaCress)	Significant reduction in thickness, pruritus, and vascularity; comparable to pressure therapy; lowers recurrence; Comparable TEWL reduction; cost‐effective options for long‐term care	Gels may have cosmetic limitations (drying time, sheen); adherence challenges with sheets; Less robust clinical data; needs further validation	ACTRN12616001100482	[138, 149, 166, 167, 168, 169, 170, 171, 172, 173, 174]
**PGT**	Sustained mechanical compression modulates collagen remodeling and scar proliferation; Incorporates feedback from pressure sensors for optimized fit and compression	Variable effectiveness; may improve thickness, elasticity, and hydration when personalized or combined with other modalities; Improved treatment precision; significant reduction in thickness and enhanced hydration and pliability after 12 weeks	Poor adherence; heterogeneity in pressure levels and protocols; inconsistent outcomes across studies; High cost; access and patient compliance may still limit widespread adoption	KCT0005918	[175, 176, 177]

### 4.3 Emerging Therapies

Emerging therapies for HTS and keloids are shifting from symptom‐based approaches toward targeting core molecular and cellular mechanisms. Gene therapy, stem cell‐based interventions, and biologics represent the forefront of innovative treatments with the potential to improve outcomes where conventional modalities fall short.

#### 4.3.1 Gene and Stem Cell–Based Therapies

Gene and stem cell therapies aim to correct dysregulated fibrotic signaling in keloids and HTS. Gene therapy focuses on modulating the expression of key fibrogenic regulators, including noncoding RNAs, whereas stem cell strategies emphasize paracrine signaling and tissue regeneration.

MicroRNA‐mediated regulation has gained particular interest. miR‐3606‐3p suppresses integrin αV (ITGAV), thereby inhibiting the integrin‐mediated FAK–Akt/ERK axis, a critical pathway in fibroblast proliferation and ECM accumulation [76]. In vivo administration of miR‐3606‐3p significantly attenuated collagen deposition and myofibroblast contractility, suggesting potent antifibrotic effects. Similarly, miR‐141‐3p, which is delivered via ADSC exosomes, targets TGF‐β2 and suppresses TGF‐β/Smad signaling, leading to decreased scar thickness and improved collagen architecture  [182, 183].

Targeting signaling molecules such as CYP24A1 and HOXC10 has also shown therapeutic promise. CYP24A1 inhibition reduces vitamin D degradation and downregulates profibrotic gene expression  [184], whereas silencing HOXC10—an oncogene overexpressed in HTS—suppresses TGF‐β‐Smad–mediated fibroblast activation  [185]. Additional gene targets, such as PLXDC2, FOXF2, and THBS2, have emerged from single‐cell transcriptomic profiling of HTS fibroblasts, further highlighting the complexity and heterogeneity of fibrotic regulation [186, 187].

Stem cell therapies, particularly those utilizing ADSC‐derived exosomes, are being explored for their anti‐inflammatory and antifibrotic effects. These exosomes modulate pathways, including the PI3K/AKT/mTOR pathway, restore mitochondrial function, and promote autophagy in keloid fibroblasts [188]. ADSC‐derived peptides such as ADSCP2 also remodel fibroblast metabolism and inhibit collagen and α‐SMA expression [189]. Combining stem cells with gene therapy—for example, by genetically enhancing mesenchymal stem cells to overexpress decorin—could offer synergistic antifibrotic benefits.

#### 4.3.2 Biologics and Antibody‐Based Interventions

Biological therapies target specific molecules involved in scar pathophysiology, offering more selective and sustained treatment options. Although systemic inhibition poses safety concerns, targeted agents such as SB431542 (an ALK5 inhibitor) have shown efficacy in preclinical models  [190].

Interleukin‐1 (IL‐1) antagonists such as anakinra have demonstrated anti‐inflammatory effects and reduced fibrosis in keloid models  [106, 191]. Similarly, antibody‐based interventions against the VEGF and FGF pathways suppress angiogenesis and fibroblast activation, limiting scar progression  [192]. MMP‐targeting antibodies aim to restore ECM homeostasis by regulating matrix degradation  [193].

An integrative approach that combines biologics with stem cell‐ or gene‐based therapies is increasingly favored. For example, pairing anti‐VEGF antibodies with MNs or laser therapy may synergize angiogenesis control with physical remodeling. These multimodal strategies have the potential to address both the inflammatory and fibrotic components of pathological scarring. Emerging molecular therapies for pathological scarring are presented in Table 5.

**TABLE 5:** Emerging molecular therapies for pathological scarring: mechanisms, pathway targets, and preclinical evidence.

Pathway	Mechanism of action	Therapeutic potential	Research models	References
**TGF‐β signaling**	Central regulator of fibrosis via canonical (Smad2/3) and noncanonical (MAPK, PI3K/Akt, Rho GTPase) cascades; enhanced by mechanical stress; crosstalk with integrin and epigenetic modulators	Anti‐TGF‐β1 antibodies, Smad pathway inhibitors (e.g., isorhamnetin), HDAC5/7 modulators (e.g., TSA), ITGB1 inhibitors (e.g., crizotinib), Fn14 antagonists	Fibroblasts on rigid substrates, stiff biomaterial‐induced mouse models	[63, 64, 65, 66, 67, 68, 69, 70, 71, 72, 73, 74]
**Integrin–FAK axis**	Mediates mechanotransduction through integrin clustering and FAK phosphorylation (Tyr397); activates PI3K/Akt, ERK, and mTOR pathways; amplifies TGF‐β responses; modulated by lumican and Snail‐1	FAK inhibitors, lumican application, Snail‐1 inhibitors	Scar‐derived fibroblasts, endothelial cells, wound‐healing mouse models	[75, 76, 77, 78, 79, 80, 81, 82, 83, 84]
**YAP/TAZ signaling**	Activated via integrin–FAK–RhoA–ROCK in response to matrix stiffness; nuclear translocation induces CTGF, COL1A1 expression; interacts with TGF‐β and Wnt/β‐catenin signaling	YAP/TAZ inhibitors, Hippo pathway restoration (e.g., RAP2 activators), Trps1 modulation for regenerative healing	HSF cultures, YAP/TAZ knockout mouse models	[85, 86, 87, 88]
**PI3K/Akt pathway**	Promotes fibroblast proliferation, angiogenesis, and ECM production; activated by mechanical cues and calcium influx; downstream of mTOR and linked to pressure stimuli	PI3K/Akt inhibitors, calcium channel blockers	HaCaT and HSFs cultured under negative pressure or mechanical strain	[89, 90, 91, 92]
**Rho GTPases (ROCK)**	Regulates cytoskeletal contractility and matrix remodeling; upregulated in tension‐induced fibrosis	ROCK inhibitors to reduce myofibroblast activation and contracture	Tension‐stimulated fibroblast cultures; ROCK inhibition studies	[93, 94]
**Ion channels (TRP)**	Mechanosensitive TRP channels mediate Ca^2^⁺ influx, promoting fibroblast tension and ECM deposition; linked to PI3K/Akt signaling	TRP channel blockers to reduce calcium‐driven myofibroblast contractility	Stretch‐induced fibroblast models	[91, 95]
**MicroRNA‐based**	miR‐3606‐3p targets ITGAV and inhibits FAK–Akt/ERK axis; miR‐141‐3p (via ADSC exosomes) suppresses TGF‐β2/Smad signaling	Antifibrotic gene modulation via miRNA mimics or exosomal delivery	In vivo wound models, ADSC cultures	[63, 64, 65, 66, 67, 68, 69, 70, 71, 72, 73, 74]
**Gene‐specific targets**	CYP24A1 inhibition decreases vitamin D degradation and profibrotic gene expression; HOXC10 silencing suppresses TGF‐β–Smad signaling	siRNA or CRISPR‐based suppression of CYP24A1 and HOXC10; potential for precise gene correction	HTS fibroblast cultures, preclinical animal models	[75, 76, 77, 78, 79, 80, 81, 82, 83, 84]
**Stem cell–derived exosomes**	ADSC exosomes modulate PI3K/Akt/mTOR, restore mitochondrial dynamics, promote autophagy; ADSCP2 peptide inhibits α‐SMA and collagen production	Exosome therapy or engineered stem cells expressing antifibrotic genes (e.g., decorin)	In vitro keloid fibroblast cultures, wound healing animal models	[85, 86, 87, 88]
**Biologics and antibodies**	Target cytokines (e.g., IL‐1), growth factors (e.g., VEGF, FGF), or MMPs to regulate inflammation, angiogenesis, and ECM remodeling	Anakinra (IL‐1 antagonist), anti‐VEGF/FGF antibodies, MMP‐targeting antibodies; potential synergy with microneedles or laser therapy	Keloid models, angiogenesis assays, preclinical combinatory studies	[89, 90, 91, 92]

## 5 Outlook

Despite considerable progress in elucidating the epidemiological patterns, cellular dynamics, and signaling mechanisms underlying HTS and keloids, effective clinical translation remains limited. These fibroproliferative disorders persist as a formidable therapeutic challenge, reflecting the complex convergence of biomechanical stress, immune dysregulation, and fibroblast heterogeneity. Although current treatment regimens—ranging from surgical excision and corticosteroid injections to silicone dressings and laser therapy—offer partial efficacy, recurrence remains common, and outcomes are unpredictable. This underscores an urgent need to move beyond reactive interventions toward a more mechanistic and individualized understanding of scar biology [194].

Notably, recent advances in fibroblast lineage tracing and single‐cell sequencing have revealed remarkable heterogeneity in scar‐forming cell populations, suggesting that pathological fibrosis is not merely a function of excessive matrix deposition but rather a dynamic interplay between profibrotic cell subsets, immune infiltration, and mechanical cues within a disrupted tissue niche. However, most therapeutic approaches continue to target bulk processes—such as ECM synthesis or inflammation—without accounting for cellular diversity or temporal changes in the fibrotic microenvironment. Future strategies must prioritize spatially and temporally resolved interventions that are tuned to the evolving biology of the scar.

In parallel, efforts to characterize the molecular architecture of HTS and keloids have revealed a dense network of interlinked signaling pathways—TGF‐β, Wnt/β‐catenin, YAP/TAZ, JAK/STAT, and Notch—each contributing to fibroblast activation, immune modulation, and matrix remodeling [195]. While these pathways have yielded promising therapeutic targets, the functional redundancy and compensatory mechanisms between them complicate single‐pathway inhibition. Moreover, despite the appeal of pathway‐specific therapies such as small‐molecule inhibitors or RNA interference, challenges in local delivery, tissue specificity, and off‐target effects continue to limit their clinical applicability. Nanotechnology‐based platforms and biomaterial‐mediated delivery systems offer potential solutions, yet these remain largely experimental and have not been validated in scar‐focused clinical trials [196].

An additional layer of complexity is introduced by the metabolic reprogramming observed in fibrotic tissues. Aberrant glycolytic flux, altered glutamine metabolism, and dysregulated lipid synthesis have been documented in both HTS and keloids, suggesting that the metabolic dimension of fibroblast activation remains poorly explored. Targeting such pathways could represent a novel antifibrotic axis distinct from canonical signal transduction mechanisms. Similarly, the immune landscape of pathological scars—characterized by persistent macrophage activation, T‐cell infiltration, and altered cytokine profiles—suggests that immunomodulatory therapies, perhaps modeled on approaches in autoimmune and neoplastic diseases, may hold untapped potential [197].

Crucially, the development of robust and translatable preclinical models remains a bottleneck in scar research. Most existing animal models inadequately recapitulate the chronicity, immune complexity, and biomechanical context of human scars [198, 199]. This limits not only mechanistic discovery but also the predictive validity of therapeutic testing. The generation of humanized or bioengineered scar models incorporating patient‐derived fibroblasts and immune components within tunable 3D scaffolds may offer a promising alternative and deserves greater investment.

The integration of omics technologies, mechanobiology, and regenerative medicine is likely to define the next chapter in scar therapeutics. Gene editing tools such as CRISPR/Cas9 offer the possibility of precisely modulating profibrotic genes—including COL1A1 and TGFBR2 [65]—although safety, delivery, and regulatory hurdles remain significant. Likewise, stem cell‐derived extracellular vesicles and bioengineered hydrogels show promise in modulating the fibrotic niche and restoring tissue architecture [200]. However, these approaches require rigorous long‐term evaluation to ensure reproducibility, safety, and functional durability in human patients.

Ultimately, progress in pathological scar management will hinge on the convergence of disciplines—bringing together molecular biology, bioengineering, immunology, and clinical dermatology. A shift toward personalized scar medicine, grounded in a patient's genetic predisposition, immune profile, and wound biomechanics, is increasingly plausible given current technological momentum [114]. However, translating this vision into clinical reality will require not only scientific innovation but also systemic reform in trial design, outcome measurement, and interdisciplinary collaboration. Moving beyond symptomatic control to achieve true scar reversal or prevention remains a formidable but increasingly tangible goal.

## Author Contributions

**Xiaowan Fang**: conceptualization, data curation, writing – original draft. **Yuxiang Wang**: conceptualization, data curation, writing – original draft. **Hao Chen**: conceptualization, data curation, writing – original draft. **Zhenzhen Yan**: formal analysis, visualization. **Shunxin Jin**: methodology, validation. **Yixin Wu**: resources, software. **Futing Shu**: supervision, writing – review and editing. **Shichu Xiao**: supervision, writing – review and editing. All authors have read and approved the final manuscript.

## Ethics Statement

The authors have nothing to report.

## Conflicts of Interest

The authors declare no conflicts of interest.

## Data Availability

The datasets generated and/or analyzed during this study are available from the corresponding author upon reasonable request. All figures included in this manuscript have obtained appropriate permissions for publication.
